# Evaluating photosynthetic models and their potency in assessing plant responses to changing oxygen concentrations: a comparative analysis of *A*_n_–*C*_a_ and *A*_n_–*C*_i_ curves in *Lolium perenne* and *Triticum aestivum*

**DOI:** 10.3389/fpls.2025.1575217

**Published:** 2025-04-29

**Authors:** Zi-Piao Ye, Xiao-Long Yang, Zi-Wu-Yin Ye, Ting An, Shi-Hua Duan, Hua-Jing Kang, Fu-Biao Wang

**Affiliations:** ^1^ New Quality Productivity Research Center, Guangdong ATV College of Performing Arts, Deqing, China; ^2^ Institute of Biophysics, Math & Physics College, Jinggangshan University, Ji’an, China; ^3^ School of Life Sciences, Nantong University, Nantong, China; ^4^ State Key Laboratory of Environmental Chemistry and Ecotoxicology, Research Center for Eco-Environmental Sciences, Chinese Academy of Sciences, Beijing, China; ^5^ School of Foreign Languages, Guangdong Baiyun University, Guangzhou, China; ^6^ College of Bioscience and Engineering, Jiangxi Agriculture University, Nanchang, China; ^7^ School of Life Sciences, Jinggangshan University, Ji’an, China; ^8^ Wenzhou Key Laboratory of Early-Maturing Tea Tree Breeding, Wenzhou Academy of Agricultural Sciences, Wenzhou, Zhejiang, China

**Keywords:** photosynthesis models, C 3 plants, FvCB model, parameter estimation, CO 2 -response to photosynthesis, apparent photorespiratory rate

## Abstract

Accurate determination of photosynthetic parameters is essential for understanding how plants respond to environmental changes. In this study, we evaluated the performance of the Farquhar-von Caemmerer-Berry (FvCB) model and introduced a novel model to fit photosynthetic rates against ambient CO_2_ concentration (*A*_n_ *–C*_a_) and intercellular CO_2_ concentration (*A*_n_ *–C*_i_) curves for *Lolium perenne* and *Triticum aestivum* under 2% and 21% O_2_ conditions. We observed significant discrepancies in the FvCB model’s fitting capacity for *A*_n_ *–C*_a_ and *A*_n_ *–C*_a_ curves across different oxygen regimes, particularly in estimates of key parameters such as the maximum carboxylation rate (*V*_cmax_), the day respiratory rate (*R*_day_), and the maximum electron transport rate for carbon assimilation (*J*_A-max_). Notably, under 2% and 21% O_2_ conditions, the values of *V*_cmax_ and *R*_day_ derived from *A*_n_ *–C*_a_ curves using the FvCB model were 46.98%, 44.37%, 46.63%, and 37.66% lower than those from *A*_n_ *–C*_i_ curves for *L. perenne*, and 47.10%, 44.30%, 47.03%, and 37.36% lower for *T. aestivum*, respectively. These results highlight that the FvCB model yields significantly different *V*_cmax_ and *R*_day_ values when fitting *A*_n_ *–C*_a_ versus *A*_n_ *–C*_i_ curves for these two C_3_ plants. In contrast, the novel model demonstrated superior fitting capabilities for both *A*_n_ *–C*_a_ and *A*_n_ *–C*_i_ curves under 2% and 21% O_2_ conditions, achieving high determination coefficients (*R* ^2^≥ 0.989). Key parameters such as the maximum net photosynthetic rate (*A*_max_) and the CO_2_ compensation point (*Γ*) in the presence of *R*_day_, showed no significant differences across oxygen concentrations. However, the apparent photorespiratory rate (*R*_pa0_) and photorespiratory rate (*R*_p0_) derived from *A*_n_ *–C*_i_ curves consistently exceeded those from *A*_n_ *–C*_a_ curves for both plant species. Furthermore, *R*_pa0_ values derived from *A*_n_ *–C*_a_ curves closely matched observed values, suggesting that *A*_n_ *–C*_a_ curves more accurately reflect the physiological state of plants, particularly for estimating photorespiratory rates. This study underscores the importance of selecting appropriate CO_2_-response curves to investigate plant photosynthesis and photorespiration under diverse environmental conditions, thereby ensuring a more accurate understanding of plant responses to changing environments

## 1 Introduction

In the context of ongoing global climate change and the persistent increase in atmospheric CO_2_ concentrations, studying the response models of plant photosynthesis to intercellular CO_2_ concentration (*C*_i_) (see **Supplementary Table S1** for the list of abbreviations) and ambient CO_2_ concentration (*C*_a_) is of critical importance. Photosynthesis, the process by which plants convert CO_2_ into organic matter using light energy, plays a crucial role in the carbon cycle and the energy flow within ecosystems (Sergio et al., 2015; Kolari et al., 2014; Roberta et al., 2024). This process is influenced by both light intensity and CO_2_ concentrations, making the development of accurate photosynthesis models essential for predicting plant growth and ecosystem changes (Eric et al., 2019; Pleban et al., 2020; Taylor et al., 2024).

The response models of photosynthesis to *C*_i_ (*A*_n_–*C*_i_ model, where *A*_n_ represents the net photosynthetic rate) and to *C*_a_ (*A*_n_–*C*_a_ model) are fundamental for understanding plant photosynthesis, each addressing different aspects. The *A*_n_–*C*_i_ model primarily describes the relationship between the internal CO_2_ concentration within plant leaves and *A*_n_. This model incorporates internal gas exchange and biochemical processes, such as the carboxylation reaction of Rubisco (Kelly et al., 2016; Ye et al., 2024). *C*_i_ denotes the CO_2_ concentration in the air space around mesophyll cells, which is influenced by stomatal conductance (Miner and Bauerle, 2017; Taylor et al., 2024). This model is typically used to analyze photosynthetic performance under various environmental conditions, including changes in light, temperature, and CO_2_ concentrations (De Kauwe et al., 2016; Yiotis et al., 2021; Xiong et al., 2022). Conversely, the *A*_n_–*C*_a_ model focuses on the relationship between ambient CO_2_ concentration and plant photosynthesis. This model evaluates the impact of changes in atmospheric CO_2_ on photosynthesis, particularly in the context of global climate change and rising CO_2_ levels (Eric et al., 2019; Qiu and Katul, 2020). It helps predict future impacts on plant growth and ecosystem dynamics. In practical applications, the *A*_n_–*C*_i_ model provides insights into the biochemical mechanisms of photosynthesis, while the *A*_n_–*C*_a_ model is more frequently used in ecological and climate change research (Kelly et al., 2016; Miner and Bauerle, 2017; Xiong et al., 2022). Both models are invaluable tools in plant physiology and global change biology.

As we explore the complex ways in which plant photosynthesis responds to variations in CO_2_ levels, a plethora of models has been developed by scientists. These models can be broadly categorized into two types: empirical and biochemical. Among the empirical models, the Michaelis-Menten (M-M) model stands out due to its foundation in enzyme kinetics, while the exponential equation model provides a mathematical framework for describing the photosynthetic response (Watling et al., 2000; Sharkey et al., 2007; Silva-Pérez et al., 2017), offering a refined method for fitting photosynthetic curves. These empirical models are utilized to fit the *A*_n_–*C*_a_ or *A*_n_–*C*_i_ curves of plants, enabling the extraction of key parameters such as the maximum net photosynthetic rate (*A*_max_), which indicates the upper limit of photosynthetic capacity, the CO_2_ compensation point with day respiratory rate (*Γ*), which reveals the CO_2_ level at which photosynthesis balances respiration, and the apparent photorespiratory rate (*R*_pa0_) at CO_2_ concentration approaching 0 μmol mol^−1^, a measure of the energy invested in photorespiration (Leuning, 1995; Medlyn et al., 2011; Morfopoulos et al., 2014). These three pivotal photosynthetic parameters are quantifiable (Medlyn et al., 2011; Morfopoulos et al., 2014; Burnett et al., 2019). Consequently, regardless of the model used and whether it is for the *A*_n_–*C*_a_ or *A*_n_–*C*_i_ curve, the parameters derived from these models should closely match the observed data without significant discrepancies. It is only under these conditions that we can deem a model to be effective. Apparently, the M-M model and the exponential equation model, though both being asymptotic functions, can’t always accurately depict net CO_2_ assimilation rates reductions beyond the TPU-limitation phase in some plant species (Watling et al., 2000; Silva-Pérez et al., 2017). Furthermore, these two models are unable to directly estimate the critical transition point from Ribulose-1,5-bisphosphate (RuBP)-limited to TPU-limited conditions (*C*_TPU_).

Among the biochemical models, the one developed by Farquhar et al. (1980) and its subsequent modifications (Harley and Sharkey, 1991) are central. The model introduced by Farazdaghi and Edwards (1988) also contributes to the field. The Farquhar model, widely known as the FvCB model, has been extensively analyzed for its biochemical mechanisms and is used to fit the *A*_n_–*C*_i_ curve of C_3_ plants (Dubois et al., 2007; Fan et al., 2011; Busch and Sage, 2017; Rogers et al., 2017; Walker et al., 2017; Yin et al., 2021). This model provides five crucial parameters: the maximum electron transport rate (*J*_A-max_) for photosynthesis, the maximum carboxylation rate (*V*_cmax_), triose phosphate utilization rate (*V*_TPU_), day respiratory rate (*R*_day_), CO_2_ compensation point (Γ_*_) in absence of *R*_day_ and mesophyll conductance (*g*_m_) (Long and Bernacchi, 2003; Norby et al., 2017; Xiao et al., 2021). Despite its extensive use in studying the photosynthetic response of C_3_ plants to environmental changes such as light, temperature, CO_2_ concentration, and nitrogen (N) nutrition (Bellasio et al., 2015; Sharkey, 2016; Busch and Sage, 2017; Anderegg et al., 2018; Cheah and The, 2020; Han et al., 2020; Yin et al., 2021; Yin and Amthor, 2024), the FvCB model has limitations. It cannot directly estimate parameters like *A*_max_, *Γ*, and *R*_pa0_, as well as is specific to C_3_ plants.

In the current research field, the *A*_n_–*C*_i_ curves of plants are typically analyzed using biochemical models to derive key photosynthetic parameters (Farquhar et al., 1980; Harley and Sharkey, 1991; Fan et al., 2011; Vijayakumar et al., 2024). However, research on fitting the *A*_n_–*C*_a_ curve is limited, probably because *C*_i_ is directly involved in photosynthetic carboxylation while ambient *C*_a_ only indirectly affects *C*_i_ (Long and Bernacchi, 2003). Moreover, there is a paucity of studies examining whether significant differences exist between important photosynthetic parameters (e.g., *A*_max_, *Γ*, and *R*_pa0_) derived from the *A*_n_–*C*_a_ and *A*_n_–*C*_i_ curves and the observed data (Busch and Sage, 2017). Similarly, the consistency of parameters such as *J*_A-max_, *V*_cmax_, Γ_*_, *V*_TPU_, and *R*_day_, obtained from fitting gas exchange data (i.e., *A*_n_–*C*_a_ and *A*_n_–*C*_i_ curves) with biochemical models, remains underexplored (Medlyn et al., 2011; Morfopoulos et al., 2014; Yin et al., 2021; Smith et al., 2023). Accurately determining these parameters is essential for understanding plant responses to ambient CO_2_ variations, evaluating carbon assimilation efficiency, and assessing adaptability to climate change. Thus, a comparative analysis of model predictions and measured data is particularly critical.

In this study, we begin with a clear and concise explanation of the FvCB model, initially introduced by Farquhar et al. in 1980. Subsequently, we introduce a new model. This model describes the CO_2_-response curve of photosynthesis and incorporates an explicit term for *R*_day_. Additionally, we also introduce another version of the model that does not explicitly define *R*_day_. Then, we showcase how to apply both the FvCB model and our newly developed model to fit *A*_n_–*C*_a_ and *A*_n_–*C*_i_ curves, respectively, in order to estimate key photosynthetic and biochemical parameters. Finally, we utilize a modeling-observation intercomparison approach to assess the photosynthetic parameters derived from *A*_n_–*C*_a_ and *A*_n_–*C*_i_ curves for both the FvCB model and our newly developed model. For the FvCB model, these parameters include *V*_cmax_, *J*_A-max_, Γ_*_, *V*_TPU_, and *R*_day_. For the new model, they encompass *A*_max_, *C*_TPU_, *Γ*, CO_2_ compensation point (Γ_*_) in the absence of *R*_day_ and *R*_pa0_. We then determine which parameters exhibit different values when derived from the *A*_n_–*C*_a_ and *A*_n_–*C*_i_ curves. Ultimately, we evaluate whether there are significant differences in the estimated parameters obtained from these two types of curves. Through this methodological exploration, we aim to provide novel insights and tools to enhance research in plant photosynthesis.

## 2 Materials and methods

### 2.1 FvCB model description

The FvCB model considers that the carbon assimilation process of C_3_ plants includes: the Rubisco enzyme activity limitation stage, the RuBP regeneration limitation stage, and the triose phosphate utilization (*V*_TPU_) limitation stage.

In the Rubisco limitation stage (Farquhar et al., 1980; Dubois et al., 2007; Sharkey et al., 2007; Miao et al., 2009; Ellsworth et al., 2015; Yin et al., 2021), the following equation is used:


(1) $$A_{\text{c}}=\frac{V_{\text{c,max}}\left(C_{\text{i}}-\Gamma_{*}\right)}{C_{\text{i}}+K_{\text{c}}\left(1+O/K_{\text{o}}\right)}-R_{\text{day}}$$

where *V*_c,max_ is the maximum velocity of the carboxylase (μmol·m^−2^·s^−1^); *C*_i_ is the intercellular CO_2_ concentration; *K*_c_ and *K*_o_ are the Michaelis-Menten constants for CO_2_ and O_2_, respectively (Farquhar et al., 1980; Silva-Pérez et al., 2017); *O* is the partial pressure of oxygen at the site of Rubisco. *R*_day_ is the day respiratory rate.

During the RuBP-limited phase of photosynthesis, if the regeneration of RuBP is predominantly constrained by the availability of NADPH, then the following equation can be applied:


(2) $$A_{\text{j}}=J\frac{C_{\text{i}}-\Gamma_{*}\;}{4C_{\text{i}}+8\Gamma_{*}}-R_{\text{day}}$$

where *A*_j_ is the carbon assimilation rate limited by RuBP regeneration capacity. At light saturation, *J* is equal to *J*_A-max_ in Equation 2 (Farquhar et al., 1980; Gu et al., 2010; von Caemmerer, 2013; Farquhar and Busch, 2017; Yin et al., 2021).

When RuBP regeneration is co-determined by the availability of both NADPH and ATP, the equation assumes a slightly modified form:


(3) $$A_{\text{j}}=J\frac{C_{\text{i}}-\Gamma_{*}\;}{4.5C_{\text{i}}+10.5\Gamma_{*}}-R_{\text{day}}$$

At light saturation, *J* is equal to *J*_A-max_ in Equation 3 (von Caemmerer, 2000; Long and Bernacchi, 2003; Yin et al., 2004; Yin et al., 2009; Lenz et al., 2010; Bernacchi et al., 2013).

In the TPU limitation stage, we have:


(4) $$A_{\text{p}}=3V_{\text{TPU}}\frac{C_{\text{i}}-\Gamma_{*}}{C_{\text{i}}-\left(1+3\alpha_{\text{G}}\right)\Gamma_{*}}-R_{\text{day}}$$

where *A*_p_ is the carbon dioxide assimilation rate when the utilization of phosphate glucose is limited. *α*_G_ is the proportion of glycolic acid not returned to chloroplasts and is related to the release of phosphate (Dubois et al., 2007). *α*_G_ is between [0, 1]. While $\;\alpha_{G}$ is equal to 0, Equation 4 is expressed as $A_{\text{p}}=3V_{\text{TPU}}-R_{\text{day}}$ (Long and Bernacchi, 2003; Bernacchi et al., 2013).

Based on the analysis above, it can be inferred that fitting the *A*_n_–*C*_a_ and *A*_n_–*C*_i_ curves separately using the FvCB model should yield different values for *V*_cmax_, *J*_A-max_, and *R*_day_, while the values for *V*_TPU_ and Γ_*_ should be the same. For *V*_TPU_ and Γ_*_, it is not possible for their values to change simply because *C*_a_ or *C*_i_ is altered. This should be consistent with the actual situation.

On the other hand, experimental methodologies facilitate the accurate quantification of whole-chain electron transport, represented by *J*_f_, through the application of fluorescence techniques. As elucidated by von Caemmerer (2000) and Long and Bernacchi (2003), *J*_f_ is partitioned among several pivotal processes, with photosynthesis being a principal recipient and denoted by *J*_A_. Beyond photosynthesis, *J*_f_ contributes to other synthetic and electron-consuming pathways, including photorespiration (*J*_O_), the reduction of nitrate to ammonium (*J*_Nit_), and the light-driven oxygen uptake via the Mehler ascorbate peroxidase (MAP) reaction (*J*_MAP_). Consequently, the relationship can be encapsulated as *J*_f_ = *J*_A_ + *J*_O_ + *J*_Nit_ + *J*_MAP_. The analysis underscores a critical correlation between *J*_f_ and *J*_A_: the magnitude of *J*_f_ must necessarily surpass that of *J*_A_. This inference arises from the understanding that *J*_f_ encompasses not only the electrons associated with *J*_A_. It also includes those in other synthetic and metabolic pathways, such as *J*_O_, *J*_Nit_, and *J*_MAP_. Consequently, it can be deduced that the maximum rate of photosynthesis (*J*_A-max_) as determined by the FvCB model must be inherently lower than the maximum rate of whole-chain electron transport (*J*_f-max_).

In recent years, significant progress has been made in the field of plant photosynthesis models. Among them, the FvCB model has also achieved remarkable advancements in aspects such as dynamic light response expansion and multi-scale coupling models. Under dynamic light environments, stomatal conductance also changes dynamically and interacts with photosynthesis. Previous studies have coupled the dynamic stomatal model with the FvCB model (Zhang et al., 2017; Yin and Amthor, 2024). They found that by using an improved version of the Ball-Berry stomatal model, the dynamic effects of factors such as light intensity, carbon dioxide concentration, and air humidity on stomatal conductance can be considered. Stomatal conductance (*g*_s_) can be expressed as: *g*_s_ = *g*_0_ + *m* × *A*_n_/*C*_a_ × *h*_s_, where *g*_0_ is the minimum stomatal conductance, *m* is an empirical coefficient, *A*_n_ is the net photosynthetic rate, *C*_a_ is the atmospheric CO_2_ concentration, and *h*_s_ is the relative humidity of the air at the leaf surface. Under dynamic light environments, changes in light intensity affect the photosynthetic rate, which in turn dynamically adjusts the stomatal conductance through the stomatal model and then feedback-affects photosynthesis. This enables the model to more realistically simulate the photosynthesis-stomatal coupling process of plants under dynamic light environments.

### 2.2 A new model for describing the CO_2_–response curve of photosynthesis with an explicit R_day_

In order to estimate key photosynthetic parameters such as *A*_max_, *C*_TPU_ and Γ_*_, we have developed a new model describing CO_2_-resposne curves of photosynthesis (*A*_n_–*C* curves) (hereafter referred to Model I). The new model can be written as follows:


(5) $$A_{\text{n}}=\alpha_{\text{c}}\frac{1-\beta_{\text{c}}C}{1+\gamma_{\text{c}}C}\left(C-\Gamma_{*}\right)-R_{\text{day}}$$

where *A*_n_ is the net photosynthetic rate; Γ_*_ is the CO_2_ compensation point in the absence of *R*_day_. *α*_c_, *β*_c_ and *γ*_c_ are three coefficients that depend on plant characteristics and environmental conditions, and they are independent of *C*. *C* can represent both ambient CO_2_ concentration (*C*_a_) and intercellular CO_2_ concentration (*C*_i_).

In addition, Equation 5 can be rearranged as:


(6) $$A_{\text{n}}=\alpha_{\text{c}}\frac{1-\beta_{\text{c}}C}{1+\gamma_{\text{c}}C}C_{\text{i}}-\alpha_{\text{c}}\frac{1-\beta_{\text{c}}C}{1+\gamma_{\text{c}}C}\Gamma_{*}-R_{\text{day}}$$

In contrast to Equation 1, the initial term represents the gross rate of photosynthesis, while the subsequent term denotes the actual rate of photorespiration (*R*_p_), assuming no contribution from *R*_day_ Equation 6. Consequently, *R*_p_ can be expressed as follows:


(7) $$R_{\text{p}}=\alpha_{\text{c}}\frac{1-\beta_{\text{c}}C}{1+\gamma_{\text{c}}C}\Gamma$$

In Equation 7, *R*_p_ will decrease with *C*. Therefore, it can be used to investigate the relationship between *R*_p_ and *C* under different environmental factors. Specially, while *C* = 0 μmol mol^−1^, *R*_p0_ = $\alpha_{\text{c}}\Gamma$ . In this scenario, theoretically, *R*_p0_ will take on two different values when either *C*_a_ or *C*_i_ is at 0 μmol mol^−1^. In practice, there is only a single value that defines the CO_2_-response curve for photosynthesis. Consequently, the *R*_p0_ value serves as a benchmark to assess the plausibility of different response types. Specifically, the proximity of the *R*_p0_ values derived from fitting *A*_n_ *–C*_a_ and *A*_n_ *–C*_i_ curves to the actual measured data is one of the criteria used to evaluate which response type is more justifiable.

Supposing that *R*_day_ approximates a constant or is independent of *C*, the first derivative of Equation 5 is expressed as follows:


(8) $$\frac{dA_{\text{n}}}{dC}=\alpha_{\text{c}}\frac{1-2\beta_{\text{c}}C-\beta_{\text{c}}\gamma_{\text{c}}C^{2}+\left(\beta_{\text{c}}+\gamma_{\text{c}}\right)\Gamma_{*}}{{\left(1+\gamma_{\text{c}}C\right)}^{2}}$$

where *dA*_n_ */dC* is the slope of the *A*_n_ *–C* curve, and *dA*_n_ */dC* decreases with increasing *C*. As *C* tends to zero in Equation 8, *dA*_n_ */dC* equals $\alpha_{\text{c}}[1+\left(\beta_{\text{c}}+\gamma_{\text{c}}\right)\Gamma_{*}]$ , and it is referred to as the initial slope of the *A*_n_ *–C* curve (*α*_0_ = $\alpha_{\text{c}}[1+\left(\beta_{\text{c}}+\gamma_{\text{c}}\right)\Gamma_{*}]$ ). When *dA*_n_ */dC* equals zero, *C*_TPU_ can be calculated, then *dA*_n_ */dC* will be negative when *C* surpasses *C*_TPU_. Therefore, Equation 5 is a non-asymptotic function.

When *dA*_n_ */dC*= 0, *C*_TPU_ can be calculated Equation 9:


(9) $$C_{\text{TPU}}=\frac{\sqrt{\left(\beta_{\text{c}}+\gamma_{\text{c}}\right)\left(1+\gamma_{\text{c}}\Gamma_{*}\right)/\beta_{\text{c}}}-1}{\gamma_{\text{c}}}$$

And *A*_max_ can be calculated Equation 10:


(10) $$A_{\text{max}}=\alpha_{\text{c}}{\left[\frac{\sqrt{\beta_{\text{c}}+\gamma_{\text{c}}}-\sqrt{\beta_{\text{c}}\left(1+\gamma_{\text{c}}\Gamma_{*}\right)}}{\gamma_{\text{c}}}\right]}^{2}-R_{\text{day}}$$

In addition, when *A*_n_ equals zero in Equation 5, *Γ* can be calculated Equation 11:


(11) $$\Gamma =\frac{\left(1+\beta_{\text{c}}\Gamma_{*}-\frac{\gamma_{\text{c}}R_{\text{day}}}{\alpha_{\text{c}}}\right)-\sqrt{{\left(1+\beta_{\text{c}}\Gamma_{*}-\frac{\gamma_{\text{c}}R_{\text{day}}}{\alpha_{\text{c}}}\right)}^{2}-4\beta_{\text{c}}\left(\frac{R_{\text{day}}}{\alpha_{\text{c}}}+\Gamma_{*}\right)}}{2\beta_{\text{c}}}$$

Indeed, in practical terms, there is but a single set of values for *A*_max_ and *Γ* that characterizes the CO_2_-response curve of photosynthesis. These values are pivotal as they provide a reference point to evaluate the reasonableness of various response types. Notably, the closeness of the *A*_max_ and *Γ* values, as determined by fitting the *A*_n_ *–C*_a_ and *A*_n_ *–C*_i_ curves, to the observed data obtained serves as a critical criterion. This comparison helps in ascertaining which response type is more rational and aligned with the actual physiological processes of photosynthesis.

### 2.3 A new model for describing the CO_2_–response curve of photosynthesis without an explicit R_day_

Since precisely measuring *R*_day_ in plants remains a challenging (Atkin and Tjoelker, 2003; Yin et al., 2009) (see Tcherkez et al., 2017 for a comprehensive review), we have developed an alternative model. It can not only accurately fit the CO_2_–response curve of photosynthesis (*A*_n_–*C* curve) but also minimize potential fitting discrepancies due to different choices of *R*_day_ (hereafter referred to Model II). The model is expressed as follows:


(12) $$A_{\text{n}}=\alpha_{c1}\frac{1-\beta_{c1}C}{1+\gamma_{c1}C}\left(C-\Gamma \right)$$

where *α*_c1_, *β*_c1_, and *γ*_c1_ are three coefficients that depend on plant characteristics and environmental conditions; *Γ* is the photorespiratory CO_2_ compensation point in presence of *R*_day_ (Farquhar et al., 1980; Long and Bernacchi, 2003). Furthermore, considering the influence of *R*_day_, it is anticipated that the coefficients of *α*_c1_, *β*_c1_, and *γ*_c1_ in Equation 12 will differ from those of *α*_c_, *β*_c_ and *γ*_c_ in Equation 5.

In addition, Equation 12 can be rearranged as:


(13) $$A_{\text{n}}=\alpha_{c1}\frac{1-\beta_{c1}C}{1+\gamma_{c1}C}C-\alpha_{c1}\frac{1-\beta_{c1}C}{1+\gamma_{c1}C}\Gamma$$

In Equation 13, the first term is the gross photosynthetic rate, and the second term is the apparent photorespiration rate (*R*_pa_) including *R*_day_. Therefore, *R*_pa_ can be expressed as:


(14) $$R_{\text{pa}}=\alpha_{c1}\frac{1-\beta_{c1}C}{1+\gamma_{c1}C}\Gamma$$

In Equation 14, *R*_pa_ decreases as *C* increases. Therefore, it can be utilized to explore the relationship between *R*_p_ and *C* for all plant species under any environmental conditions. Indeed, when *C* is exactly 0 μmol mol^−1^, *R*_pa0_ takes on a particular value. Theoretically, one might expect *R*_pa0_ to have two different values corresponding to either *C*_a_ or *C*_i_ being at 0 μmol mol^−1^. However, in actuality, there is but a single value that delineates the CO_2_-response curve for photosynthesis. This singular *C*_a_ or *C*_i_ value thus becomes an essential criterion for assessing the plausibility of various response types. Notably, the closeness of the *R*_pa0_ values, derived from fitting *A*_n_ *–C*_a_ and *A*_n_ *–C*_i_ curves, to the observed data is a pivotal factor in judging which response type is more reasonable.

The first derivative of Equation 12 may be expressed as follows:


(15) $$\frac{dA_{\text{n}}}{dC}=\alpha_{\text{c}1}\frac{1-2\beta_{\text{c}1}C-\beta_{\text{c}1}\gamma_{\text{c}1}C^{2}+\left(\beta_{\text{c}1}+\gamma_{\text{c}1}\right)\Gamma }{{\left(1+\gamma_{\text{c}1}C\right)}^{2}}$$

where *dA*_n_ */dC* is the slope of the *A*_n_ *–C* curve, and *dA*_n_ */dC* decreases with increasing *C*. As *C* tends to zero in Equation 15, *dA*_n_ */dC* equals $\alpha_{c1}[1+\left(\beta_{c1}+\gamma_{c1}\right)\Gamma_{*}]$ , and it is referred to as the initial slope of the *A*_n_ *–C* curve (i.e., *α*_0_ = $\alpha_{c1}[1+\left(\beta_{c1}+\gamma_{c1}\right)\Gamma_{*}]$ ). *dA*_n_ */dC* equals zero when *C* equals to *C*_TPU_, then *dA*_n_ */dC* will be negative when *C* surpasses *C*_TPU_. It is important to acknowledge that the new model, distinguishes between two distinct values for the *A*_n_ *–C* curve: one is *C*_i,TPU_, which represents *A*_n_ *–C*_i_, and the other is *C*_a,TPU_, which corresponds to the *A*_n_ *–C*_a_ curve. This distinction is crucial for accurately modeling and understanding the photosynthetic responses in plants.

Therefore, while the $dA_{\text{n}}/dC=0$ , *C*_TPU_ is calculated by:


(16) $$C_{\text{TPU}}=\frac{\sqrt{\left(\beta_{\text{c}1}+\gamma_{\text{c}1}\right)\left(1+\gamma_{\text{c}1}\Gamma \right)/\beta_{\text{c}1}}-1}{\gamma_{\text{c}1}}$$

And *A*_max_ can be obtained as:


(17) $$A_{\text{max}}=\alpha_{\text{c}1}{\left[\frac{\sqrt{\beta_{\text{c}1}+\gamma_{\text{c}1}}-\sqrt{\beta_{\text{c}1}\left(1+\gamma_{\text{c}1}\Gamma \right)}}{\gamma_{\text{c}1}}\right]}^{2}-R_{\text{day}}$$

Besides *C*_TPU_ and *A*_max_ can be calculated by Equations 16, 17, respectively, *Γ* can also be estimated by Equation 12. Indeed, in practical applications, there is but one definitive set of values for *A*_max_ and *Γ* that characterizes the CO_2_-response curve of photosynthesis. These values are crucial as they serve as a benchmark against which the reasonableness of different response types can be assessed. Importantly, the proximity of the *A*_max_ and *Γ* values, derived from fitting the *A*_n_ *–C*_a_ and *A*_n_ *–C*_i_ curves, to the actual observed data is a key criterion. This comparison is essential for determining which response type is more rational and in accordance with the true physiological mechanisms of photosynthesis.

Furthermore, it is important to acknowledge that Equations 5, 12 are fundamentally equivalent when it comes to determining photosynthetic parameters like *A*_max_ and *Γ*, irrespective of whether *A*_n_–*C*_a_ curves or *A*_n_–*C*_i_ curves are being fitted.

### 2.4 Plant materials

In this study, two typical C_3_ plants, namely *Lolium perenne* L. (Zhongxin 830) and *Triticum aestivum* L. (Jimai 22), were selected as the experimental materials. As two important crops, their photosynthetic characteristics display the typical carbon assimilation process of C_3_ plants. Their photosynthesis is highly sensitive to environmental conditions such as light intensity, carbon dioxide concentration, and water supply (Höglind et al., 2011; Pshenichnikova et al., 2019). Therefore, these two crops are highly suitable for a comparative study of the differences between the FvCB model (applicable only to C_3_ plants) and the new model in fitting the *A*_n_–*C*_a_ and *A*_n_–*C*_i_ curves and estimating key photosynthetic parameters under different oxygen concentration conditions. The two experimental materials were sown in mid-October 2022 and managed conventionally in the field. Data were collected on sunny days from April 28 to May 10, 2023. *T. aestivum* was at the booting stage to the initial flowering stage, with a plant height of 60–70 cm, and its flag leaf was selected for measurement. *L. perenne* was at the booting stage, with a plant height of about 1.3 m, and the first leaf below its flag leaf was selected for measurement.

### 2.5 Gas exchange data measurement

A portable photosynthesis system (LI-6400-40, LI-COR INC., USA) was used to collect data on sunny days from 9:00 to 11:30 and 14:00 to 17:00, with air temperatures at 30.3 ± 2.5°C and maximum midday light intensity of about 1,600 μmol·m^-2^·s^-1^. After a 1.5- to 2-hour induction under natural light, the device was turned on for preheating and checked. The oxygen concentration in the fluorescence leaf chamber is controlled by connecting external gas cylinders filled with different gas mixtures. The oxygen concentrations are set at 2% (2% oxygen and 98% nitrogen; under this concentration, the plant’s photorespiration can be neglected, and this serves as the treatment group) and 21% (21% oxygen and 79% nitrogen; this is the atmospheric oxygen concentration, and it serves as the control group). The high-pressure gas cylinders are first connected to a self-made buffer bag. A small amount of water is injected into the buffer bag to simulate the relative humidity in the atmosphere. After passing through the buffer bag, the mixed gas enters the leaf chamber through the intake pipe of the photosynthesis instrument, ensuring the stability of the oxygen concentration and appropriate humidity. Currently, this buffer device has been granted a Chinese national utility model patent (ZL 2015 2 0174847.1). The CO_2_ injection system was calibrated using an open gas path with a flow rate of 500 μmol·s^-1^. Before measuring the *A*_n_–*C*_a_ and *A*_n_–*C*_i_ curves of the two plants, the light response curves of photosynthesis (*A*_n_–*I* curves, where *I* is light intensity) for these two plants were measured first. When measuring the *A*_n_–*I* curve, the CO_2_ injection system provided a stable CO_2_ concentration. Based on measurements of atmospheric CO_2_ concentration, the CO_2_ concentration in the instrument chamber was set to 420 μmol·mol^-1^, and the light intensity gradient was set to: 2,000, 1,800, 1,600, 1,400, 1,200, 1,000, 800, 600, 400, 200, 150, 100, 80, 50, 0 μmol·m^-2^·s^-1^. All measurements used an automatic measurement program, simultaneously recording leaf gas exchange and chlorophyll fluorescence parameters. During automatic measurement, the minimum waiting time for each recording was 2 minutes, and the maximum waiting time was 3 minutes. Before data recording, the instrument automatically performed matching between the reference chamber and the sample chamber. After the data were measured, the “Photosynthesis Model Fitting Software (PMSS)” at http://photosynthetic.sinaapp.com/calc.html (Shenzhen Baoying Technology Computing Co., Ltd., China) was used to fit the *A*_n_–*I* curves of the two plants in accordance with the photosynthesis light-response model (Ye et al., 2013). The saturated light intensity for both plants was found to be 2,000 μmol·m^-2^·s^-1^.

While measuring the *A*_n_–*C*_a_ and *A*_n_–*C*_i_ curves of the two plants, the light intensity was set to 2,000 μmol·m^-2^·s^-1^, and the O_2_ concentrations were 2% and 21%, respectively. The CO_2_ injection system provided different external CO_2_ gradients (*C*_a_): 1,600, 1,400, 1,200, 1,000, 800, 600, 420, 300, 200, 100, 50, and 0 μmol·mol^-1^. All measurements used an automatic measurement program, simultaneously recording leaf gas exchange and chlorophyll fluorescence parameters. During the process of automatic measurement, the waiting time for each recording ranged from a minimum of 2 minutes to a maximum of 3 minutes. Before data recording, the instrument automatically performed matching between the reference chamber and the sample chamber. For the FvCB model, the *A*_n_–*C*_i_ curves of the two plant species were meticulously fitted using a method developed by Sharkey et al. (2007) to estimate parameters such as *V*_cmax_, *J*_A-max_, *V*_TPU_, Γ_*_, and *R*_day_. In particular, the *R*_day_ value is derived under the assumption that it equals 0.015 *V*_cmax_. This method developed by Sharkey et al. is integrated into the Photosynthesis Model Simulation Software (PMSS) platform, which can be accessed at http://photosynthetic.sinaapp.com/calc.html (Shenzhen Baoying Technology Computing Co., Ltd., China). Moreover, to determine the *J*_f-max_, which represents the maximum electron transport rate associated with Photosystem II (PSII), a comprehensive analysis of the *J*–*C*_i_ curves obtained though measurements is necessary. The *J*_f-max_, identified as the uppermost threshold of electron transport rate within these curves, is a crucial parameter for assessing the photosynthetic potential of plants. Its significance becomes particularly pronounced after the *J*–*C*_i_ curves have been meticulously quantified and analyzed.

Additionally, the parameters *A*_max_, *C*_TPU_, *Γ*, *R*_pa0_, *R*_p0_, Γ_*_, and *R*_p_ can be determined using Model I and Model II with the assistance of the PMSS platform. The PMSS platform offers a user-friendly interface for conducting simulations and extracting key parameters that are essential for analyzing the photosynthetic performance of plants. It is a valuable tool for researchers and students alike. Please be aware that there might be temporary network issues that could prevent the webpage from loading.

### 2.6 Statistical analysis

All variables were presented as mean values and standard error (mean ± standard error, *n* = 3) with three replicates. Data were analyzed with one-way analysis of variance (ANOVA). A paired-sample *t*-test was used to compare whether there was a significant difference between the fitting results and the corresponding observed values at the 5% significance level (*p* < 0.05). The data analysis was performed using the SPSS 18.5 statistical software package (SPSS, Chicago, Illinois, USA). One paired-sample *t* test was employed to compare whether there were significant differences between the fitting results and corresponding observed values at the 5% level of significance (*p* < 0.05) using the statistical package of SPSS 18.5 (SPSS, Chicago, IL, United States). Graphs were created using Origin 2021, and final graphic processing was performed with Adobe Illustrator CS5. The determination coefficient (*R* ^2^) was used to indicate the degree of fit between the model and observed points, which was calculated as *R* ^2^ = 1 – SSE/SST, where SST is the total sum of squares and SSE is the sum of squared errors.

## 3 Results

### 3.1 A_n_–C_a_ and A_n_–C_i_ curves and their fitting with the FvCB model at 21% O_2_ concentration

**Figure 1:**
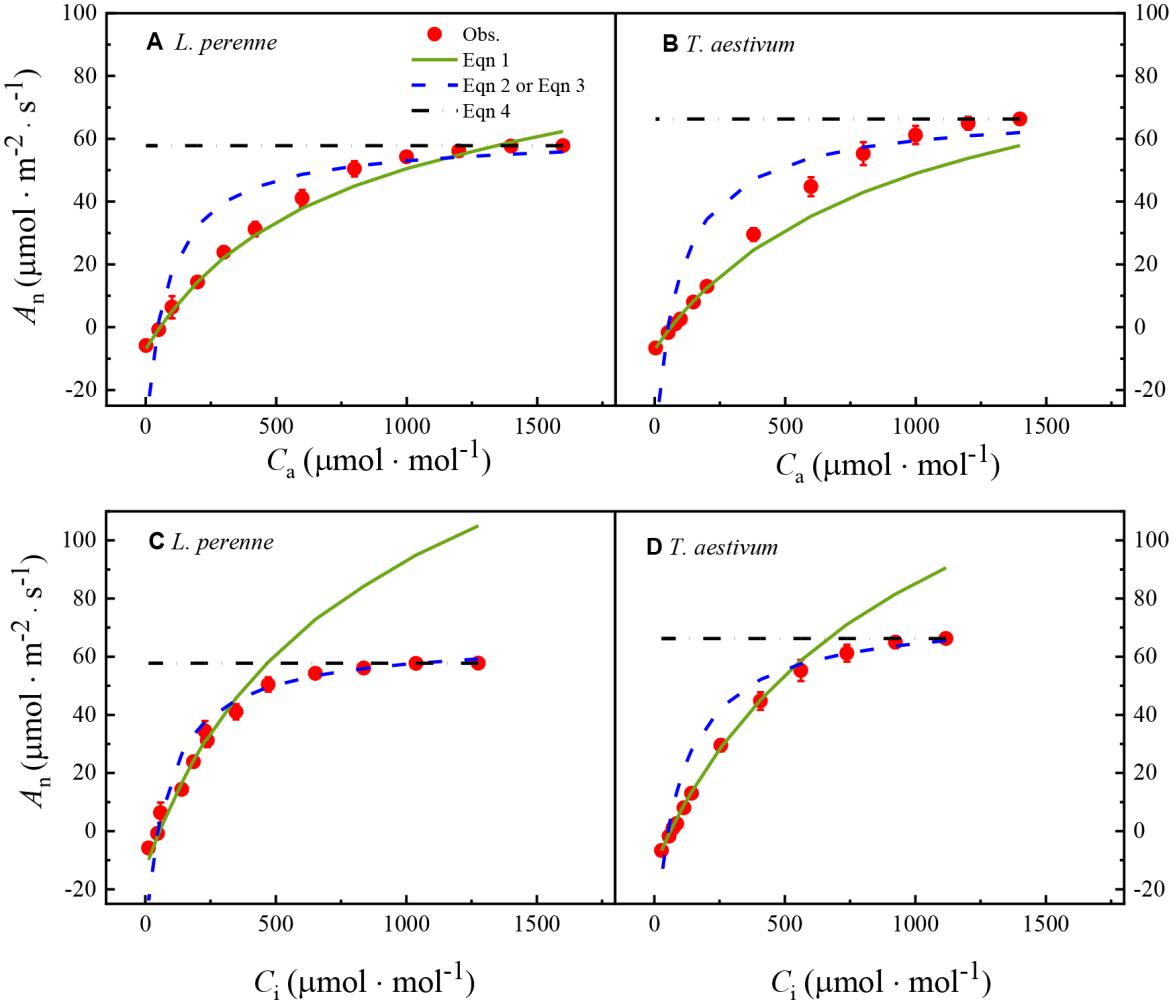
The *A*_n_–*C*_a_ and *A*_n_–*C*_i_ curves for *L. perenne* and *T.aestivum* under atmospheric conditions with an oxygen concentration of 21%. The curves have been fitted with the FvCB model, a comprehensive model that describes the photosynthetic process in C_3_ plants, taking into account the carboxylation efficiency of Rubisco, the rate of electron transport, and the triose phosphate utilization. The solid red dots on the curves represent the observed experimental data, which are the actual measurements obtained from the plants under controlled conditions. Each data point is expressed as the mean ± standard error (SE), and the experiments were conducted with three replicates (*n* = 3) to ensure the robustness and reproducibility of the results.

**Figure 1** shows the *A*_n_–*C*_a_ curves and *A*_n_–*C*_i_ curves of *L. perenne* and *T. aestivum* under the conditions of 21% O_2_ concentration. As analyzed in **Figure 1**, the FvCB model shows significant differences when fitting the *A*_n_–*C*_a_ curves and *A*_n_–*C*_i_ curves of these two plants.

**Figure 2:**
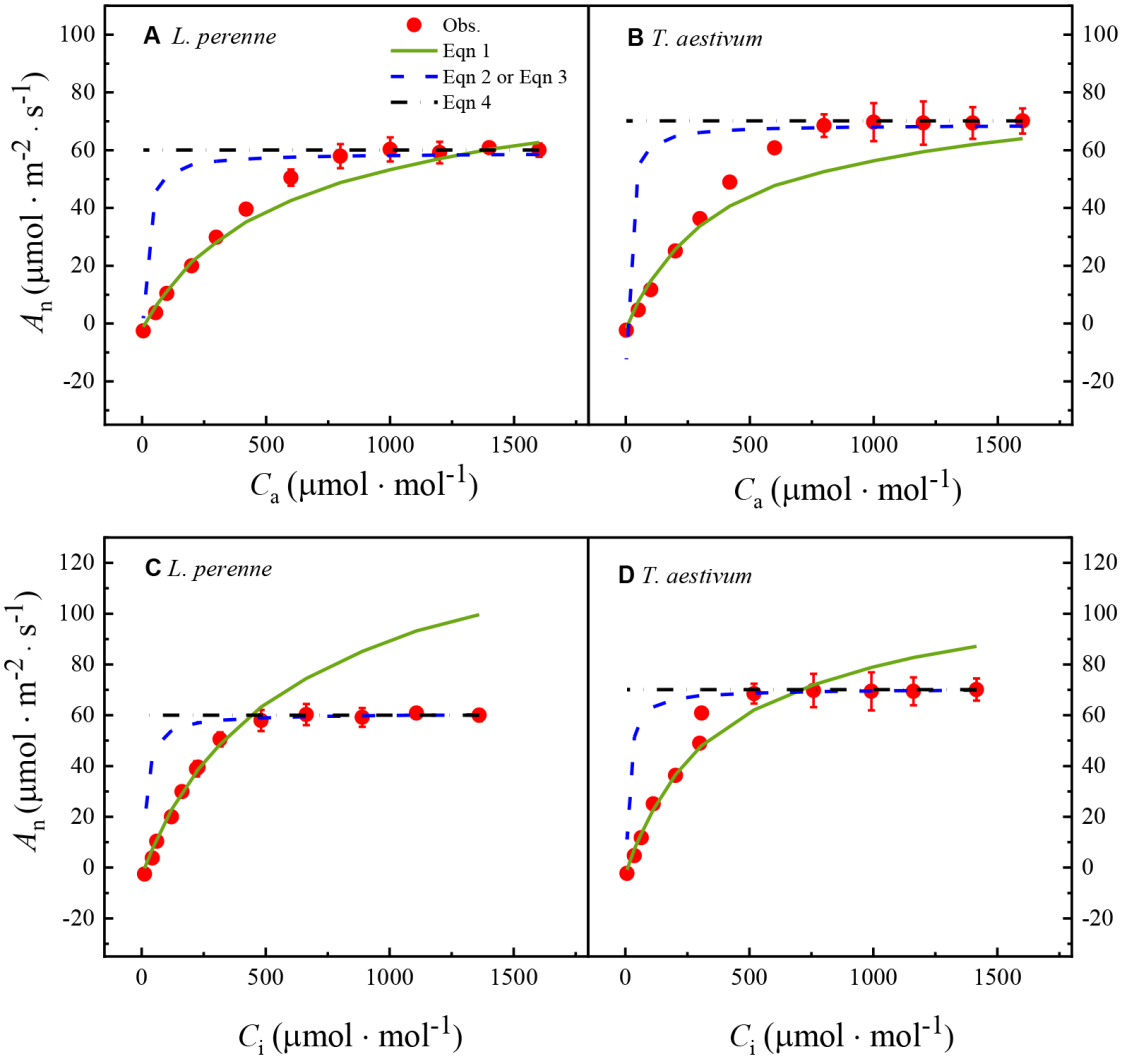
The *A*_n_–*C*_a_ and *A*_n_–*C*_i_ curves for *L. perenne* and *T. aestivum* under a reduced oxygen concentration of 2%. The curves have been fitted with the FvCB model, a comprehensive model that describes the photosynthetic process in C_3_ plants, taking into account the carboxylation efficiency of Rubisco, the rate of electron transport, and the triose phosphate utilization. The solid red dots on the curves represent the observed experimental data, which are the actual measurements obtained from the plants under controlled conditions. Each data point is expressed as the mean ± standard error (SE), and the experiments were conducted with three replicates (*n* = 3) to ensure the robustness and reproducibility of the results.

Specifically, (1) after fitting the *A*_n_–*C*_a_ curve of *T. aestivum* with Equations 1, 2, the obtained curve has a large deviation from the actual observed data (**Figure 1B**); (2) as showed in **Figure 1A**, the transition point from Rubisco limitation to RuBP limitation (*C*_i,tr_) is approximately 1,200 μmol·mol^-1^, which is significantly higher than the currently accepted empirical range of 300-600 μmol·mol^-1^, while the curve of *T. aestivum* does not show *C*_i,tr_ (**Figure 1B**), indicating a significant discrepancy from the theory that *C*_i,tr_ must exist. Furthermore, when the FvCB model fits the *A*_n_–*C*_i_ curves of the two plants, it reveals three key processes affecting C_3_ plant carbon assimilation: Rubisco limitation, RuBP regeneration limitation, and TPU limitation (**Figures 1C, D**).

**Table 1** shows the key parameters obtained by fitting the *A*_n_–*C*_a_ curves and *A*_n_–*C*_i_ curves of *L. perenne* and *T. aestivum* with the FvCB model, including *J*_A-max_, *V*_cmax_, *V*_TPU_, Γ_*_, and *R*_day_. According to the results in **Table 1**, the *V*_cmax_ and *V*_TPU_ predicted by the FvCB model are two indirect parameters that currently cannot be directly measured by experiments; Γ_*_ and *R*_day_ are two parameters that are difficult to accurately measure in experiments, so there are no corresponding observed values in this study. Among these parameters, only the model predicted value of *J*_max_ can be directly compared with the actual observed value.

**Table 1:** Observed data and results estimated by FvCB model for two C_3_ species at 21% O_2_ concentration (mean ± *SE*, *n* = 3).

	*L. perenne*					*T. aestivum*				PD (%)
	*A*_n_ *-C*_a_		*A*_n_ *-C*_i_		PD (%)	*A*_n_ *-C*_a_		*A*_n_ *-C*_i_		
	FvCB model	Obs.	FvCB model	Obs.		FvCB model	Obs.	FvCB model	Obs.	
*V*_cmax_	91.52 ± 6.89^b^	–	172.63 ± 10.57^a^	–	46.98	110.63 ± 4.53^b^	–	198.85 ± 3.23^a^	–	44.37
*J*_A-max_	246.39 ± 3.55^b^	283.85 ± 3.36^a^	276.18 ± 7.20^a^	283.85 ± 3.36^a^	10.79	283.86 ± 6.66^a^	293.78 ± 3.13^a^	316.53 ± 5.42^a^	293.78 ± 3.13^b^	10.32
*V*_TPU_	19.72 ± 0.32^a^	–	20.13 ± 0.34^a^	–	2.04	22.65 ± 0.33^a^	–	23.78 ± 0.35^a^	–	4.75
Γ_*_	41.77 ± 2.69^a^	–	41.77 ± 2.69^a^	–	0	22.39 ± 0.98^a^	–	20.54 ± 0.81^a^	–	9.01
*R*_day_	1.37 ± 0.10^b^	–	2.59 ± 0.16^a^	–	47.10	1.66 ± 0.07^b^	–	2.98 ± 0.05^a^	–	44.30
*R* ^2^	0.991	–	0.986	–	0.51	0.987	–	0.999	–	1.20

Estimated and observed parameter values within one plant which are statistically significant different (*p* < 0.05) are annotated with different superscript letter (e.g. 283.85 ± 3.36^a^ and 246.34 ± 5.48^b^ indicates a significant difference), whereas those which are not statistically significant different (*p* > 0.05) are annotated with the same superscript letter (e.g. 276.18 ± 7.20^a^ and 283.85 ± 3.36^a^ indicates no significant difference). Percentage differences (PD, %) = (Value derived from *A*_n_ *–C*_i_ curves *–* Value derived from *A*_n_–*C*_a_ curves) / Value derived from *A*_n_ *–C*_i_ curves × 100%. The PD value is represented by its absolute value. The unit of *V*_cmax_, *J*_A-max_, *V*_TPU_, and *R*_day_ is μmol m^-2^ s^-1^; the unit of Γ_*_ is μmol mol^-1^.

However, the data presented in **Table 1** indicate that the *J*_A-max_ values derived from fitting the *A*_n_–*C*_a_ curve of *L. perenne* using the FvCB model is considerably lower than the observed *J*_f-max_ value. In contrast, the *J*_A-max_ value obtained by fitting the *A*_n_–*C*_i_ curve of *L. perenne* aligns closely with the observed *J*_f-max_ value, with no significant discrepancy between the estimated and observed data. Conversely, for *T. aestivum*, the *J*_A-max_ value obtained from fitting the *A*_n_–*C*_a_ curve is close to the observed *J*_f-max_ value.

In addition, the results in **Table 1** show that the *V*_cmax_ values obtained by fitting the *A*_n_–*C*_a_ curves with the FvCB model are smaller than the values obtained by fitting the *A*_n_–*C*_i_ curves with the model by 46.98% and 44.37% for *L. perenne* and *T. aestivum*, respectively. A similar pattern is observed for the estimation of *R*_day_. That is, the *R*_day_ values obtained by fitting the *A*_n_–*C*_a_ curves with the FvCB model are significantly smaller than those obtained by fitting the *A*_n_–*C*_i_ curves with the same model, with reductions of 47.10% and 44.30% for *L. perenne* and *T. aestivum*, respectively.

### 3.2 A_n_–C_a_ and A_n_–C_i_ curves and their fitting with the FvCB model at 2% O_2_ concentration

**Figure 2** shows the *A*_n_–*C*_a_ curves and *A*_n_–*C*_i_ curves of *L. perenne* and *T. aestivum* under the conditions of 2% O_2_ concentration. As the data presented in **Figure 2**, it can be observed that the fitted curves using the FvCB model show significant differences in the values of *C*_i,tr_. Specifically, the FvCB model predicts significantly higher *C*_i,tr_ values when fitting the *A*_n_–*C*_a_ curves of *L. perenne* and *T. aestivum* compared to when fitting their *A*_n_–*C*_i_ curves (**Figure 2**). Furthermore, the fitting results of the FvCB model reveal three key biochemical processes affecting C_3_ plant carbon assimilation: Rubisco enzyme limitation process, RuBP regeneration limitation, and TPU limitation. These processes play a crucial role in plant photosynthesis under varying oxygen concentration conditions (**Figures 2C, D**). At 2% O_2_ concentration, these limitation processes may differ from those at 21% O_2_ concentration, possibly due to plant’s adaptive regulation in low-oxygen environments. For example, Rubisco enzyme limitation may be more prominent as oxygen competes with CO_2_ for Rubisco’s active site, and this competition may be intensified in low-oxygen conditions. At the same time, the regeneration of RuBP and TPU limitation may also be affected by the reduction of oxygen concentration, thereby affecting the overall efficiency of photosynthesis.

**Table 2** shows the key parameters obtained by fitting the *A*_n_–*C*_a_ and *A*_n_–*C*_i_ curves of *L. perenne* and *T. aestivum* with the FvCB model, including *J*_A-max_, *V*_cmax_, *V*_TPU_, Γ_*_, and *R*_day_. Similar to the results in **Table 1**, the four parameters *V*_cmax_, *V*_TPU_, Γ_*_, and *R*_day_ have no corresponding observed values. Only the model-predicted value of *J*_A-max_ can be directly compared with the observed *J*_f-max_ value. Furthermore, as showed in **Table 2**, regardless of whether it is the *A*_n_–*C*_a_ curves or the *A*_n_–*C*_i_ curves of *L. perenne* and *T. aestivum* fitted with the FvCB model, the derived *J*_A-max_ values closely approximate the observed *J*_f-max_ value, and there is no significant difference (*p* > 0.05) between the estimated values and the observed data (**Table 2**).

**Table 2:** Observed data and results estimated by FvCB model for two C_3_ species at 2% O_2_ concentration (mean ± *SE*, *n* = 3).

	*L. perenne*					*T. aestivum*				PD (%)
	*A*_n_ *-C*_a_		*A*_n_ *-C*_i_		PD (%)	*A*_n_ *-C*_a_		*A*_n_ *-C*_i_		
	FvCB model	Obs.	FvCB model	Obs.		FvCB model	Obs.	FvCB model	Obs.	
*V*_cmax_	71.67 ± 5.56^b^	–	134.29 ± 11.22^a^	–	46.63	72.48 ± 7.14^b^	–	116.27 ± 8.52^a^	–	37.66
*J*_A-max_	240.08 ± 6.55^a^	246.34 ± 5.48^a^	251.78 ± 6.71^a^	246.34 ± 5.48^a^	4.65	279.63 ± 11.75^a^	252.46 ± 6.90^a^	289.66 ± 14.38^a^	252.46 ± 6.90^a^	3.46
*V*_TPU_	20.37 ± 0.42^a^	–	20. 68 ± 0.40^a^	–	1.50	23.74 ± 0.87^a^	–	23.96 ± 0.87^a^	–	0.92
Γ_*_	4.42 ± 0.31^a^	–	4.42 ± 0.31^a^	–	0	3.84 ± 0.21^a^	–	3.84 ± 0.21^a^	–	0
*R*_day_	1.07 ± 0.08^b^	–	2.02 ± 0.17^a^	–	47.03	1.09 ± 0.11^b^	–	1.74 ± 0.13^a^	–	37.36

Estimated and observed parameter values within one plant which are statistically significant different (*p* < 0.05) are annotated with different superscript letter (e.g. 272.39 ± 4.89^a^ and 246.34 ± 5.48^b^ indicates a significant difference), whereas those which are not statistically significant different (*p* > 0.05) are annotated with the same superscript letter (e.g. 20.70 ± 0.41^a^ and 20.70 ± 0.41^a^ indicates no significant difference). Percentage differences (PD, %) = (Value derived from *A*_n_ *–C*_i_ curves *–* Value derived from *A*_n_–*C*_a_ curves) / Value derived from *A*_n_ *–C*_i_ curves × 100%. The PD value is represented by its absolute value. The unit of *V*_cmax_, *J*_A-max_, *V*_TPU_, and *R*_day_ is μmol m^-2^ s^-1^; the unit of Γ_*_ is μmol mol^-1^.

In addition, the results in **Table 2** also show that the *V*_cmax_ values obtained by fitting the *A*_n_–*C*_a_ curves with the FvCB model are significantly smaller (*p* < 0.05) than the values obtained by fitting the *A*_n_–*C*_i_ curves with the model by 46.63% and 37.66% for *L. perenne* and *T. aestivum*, respectively. A similar situation applies to the estimation of *R*_day_. That is, the *R*_day_ values derived by fitting the *A*_n_–*C*_a_ curves with the FvCB model are significantly lower (*p* < 0.05) than those obtained by fitting the *A*_n_–*C*_i_ curves with the same model, with reductions of 47.03% and 37.36% for *L. perenne* and *T. aestivum*, respectively. This discrepancy underscores the importance of the curve types in fitting, as it can influence estimated parameters and, in turn, our understanding of the photosynthetic responses in different plant species. Furthermore, the Γ_*_ values estimated by the FvCB model for both plant species appear to be underestimated (**Table 2**).

### 3.3 A_n_–C_a_ and A_n_–C_i_ curves and their fitting using the Model I or Model II at 21% O_2_ concentration

**Figure 3:**
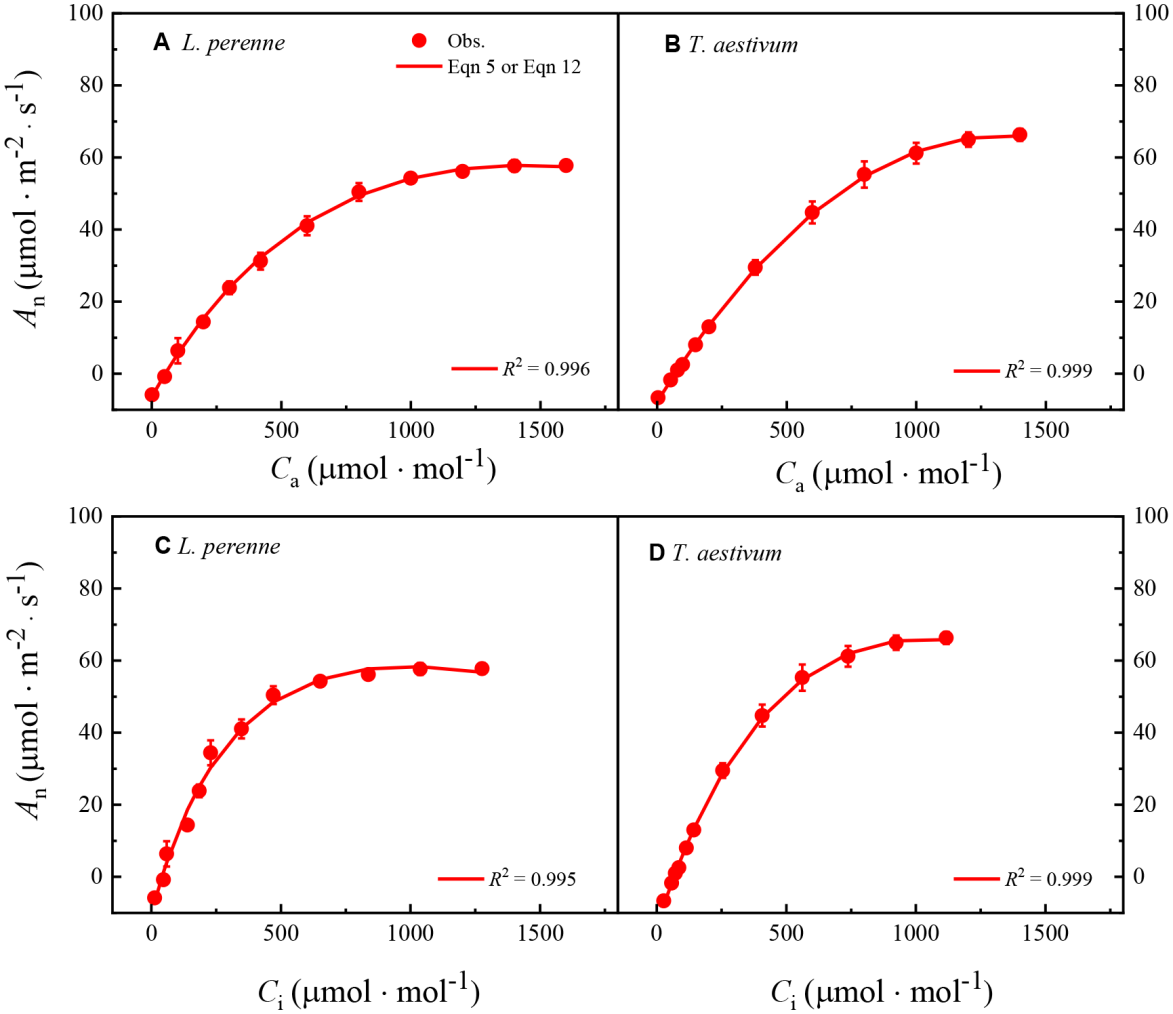
The *A*_n_–*C*_a_ and *A*_n_–*C*_i_ curves of *L. perenne* and *T. aestivum* at an ambient oxygen concentration of 21%. These curves are derived from the photosynthetic response to varying carbon dioxide concentrations, which is a critical parameter in understanding plant carbon assimilation capabilities. The curves have been precisely fitted using either Equations 5 or 12, which are two new models designed to capture the relationship between photosynthesis, internal and external carbon dioxide concentrations. The solid red dots scattered across the graphs represent the observed experimental data points, each carefully measured to ensure accuracy and reliability. These data points are presented as the mean value ± standard error (SE), with each measurement being replicated three times (*n* = 3) to ensure statistical significance and to account for biological variability.

**Figure 3** shows the *A*_n_–*C*_a_ and *A*_n_–*C*_i_ curves of *L. perenne* and *T. aestivum*, along with the fitting curves generated by Model I or Model II. As shown in **Figure 3**, both models can accurately reproduce these plant’s *A*_n_–*C*_a_ and *A*_n_–*C*_i_ curves at 21% O_2_ concentration, with the determination coefficient (*R* ^2^) at least 0.995.

**Table 3** presents several parameters derived from fitting the *A*_n_–*C*_a_ and *A*_n_–*C*_i_ curves of *L. perenne* and *T. aestivum* using Model I or Model II, including *α*_0_, *A*_max_, *C*_i,TPU_, Γ_*_, *Γ*, *R*_p0_, and *R*_pao_. The data in **Table 3** indicate that the *A*_max_ and *Γ* values obtained by fitting the *A*_n_–*C*_a_ curves of *L. perenne* and *T. aestivum* with both models do not significantly differ (*p* > 0.05) from those obtained from the *A*_n_–*C*_i_ curves. The *R*_pa0_ values derived from fitting the *A*_n_–*C*_a_ curves of *L. perenne* and *T. aestivum* with Model I or Model II are close to their respective observed values, with no significant difference (*p* > 0.05) between them (**Table 3**). However, the *R*_pa0_ values obtained by fitting the *A*_n_–*C*_i_ curves of *L. perenne* and *T. aestivum* with either Model I or Model II are considerably higher than the corresponding observed values, exhibiting a significant discrepancy (*p* < 0.05, **Table 3**). Furthermore, for *L. perenne* and *T. aestivum* at 21% O_2_ conditions, the values of *α*_0_ derived from *A*_n_ *–C*_a_ curves estimated by Model I were 57.09% and 52.26% lower than those obtained by fitting their *A*_n_–*C*_i_ curves (**Table 3**).

**Table 3:** The observed data and the outcomes generated by Model I and Model II for the two C_3_ species at a 21% O_2_ concentration for *A*_n_ *-C*_a_ and *A*_n_ *-C*_i_ curves are presented (mean ± *SE*, *n* = 3).

	*L. perenne*					*T. aestivum*				PD (%)
	*A*_n_ *-C*_a_		*A*_n_ *-C*_i_		PD (%)	*A*_n_ *-C*_a_		*A*_n_ *-C*_i_		
	Model I	Obs.	Model I	Obs.		Model I	Obs.	Model I	Obs.	
*α*_0_	0.127 ± 0.007^b^	^-^	0.296 ± 0.018^a^	^-^	57.09	0.116 ± 0.004^b^	^-^	0.243 ± 0.007^a^	^-^	52.26
*A*_max_	58.12 ± 0.73^a^	57.80 ± 0.88^a^	58.66 ± 0.61^a^	57.80 ± 0.88^a^	0.92	66.44 ± 1.15^a^	66.28 ± 0.98^a^	66.29 ± 1.14^a^	66.28 ± 0.98^a^	0.23
*C*_i,TPU_	1441.12 ± 87.03^a^	1533.26 ± 66.72^a^	908.91 ± 36.60^a^	1075.66 ± 120.47^a^	58.55	1357.05 ± 63.31^a^	1399.91 ± 0.16^a^	1006.74 ± 29.46^a^	1072.49 ± 32.85^a^	34.80
*Γ*	49.91 ± 8.35^a^	55.93 ± 4.63^a^	47.85 ± 8.39^a^	53.75 ± 4.22^a^	4.31	69.29 ± 1.92^a^	69.36 ± 1.63^a^	66.52 ± 0.37^a^	66.83 ± 0.48^a^	4.16
Γ_*_	38.76 ± 7.39^a^	–	31.03 ± 5.86^a^	–	24.91	40.24 ± 2.09^b^	–	51.57 ± 1.13^a^	–	21.97
*R*_p0_	3.88 ± 0.68^b^	–	10.79 ± 2.20^a^		64.04	4.50 ± 0.40^b^	–	11.68 ± 0.07^a^	–	61.47
*R*_day_	2.59 ± 0.16^a^	–	2.59 ± 0.16^a^	^-^	0	3.09 ± 0.08^a^	–	3.09 ± 0.08^a^	–	0
*R* ^2^	0.996	–	0.995	–	0.10	0.999	–	0.999	–	0
	A_n_-C_a_		A_n_-C_i_		PD (%)	A_n_-C_a_		A_n_-C_i_		PD (%)
	Model II	Obs.	Model II	Obs.		Model II	Obs.	Model II	Obs.	
*α*_0_	0.127 ± 0.007^b^	^-^	0.296 ± 0.018^a^	^-^	57.09	0.116 ± 0.004^b^	^-^	0.243 ± 0.007^a^	^-^	52.26
*A*_max_	58.12 ± 0.73^a^	57.80 ± 0.88^a^	58.66 ± 0.61^a^	57.80 ± 0.88^a^	0.92	66.44 ± 1.15^a^	66.28 ± 0.98^a^	66.29 ± 1.14^a^	66.28 ± 0.98^a^	0.23
*C*_i,TPU_	1441.12 ± 87.03^a^	1533.26 ± 66.72^a^	908.91 ± 36.60^a^	1075.66 ± 120.47^a^	58.55	1357.05 ± 63.31^a^	1399.91 ± 0.16^a^	1006.74 ± 29.46^a^	1072.49 ± 32.85^a^	34.80
*Γ*	49.91 ± 8.35^a^	55.93 ± 4.63^a^	47.85 ± 8.39^a^	53.75 ± 4.22^a^	4.31	69.29 ± 1.92^a^	69.36 ± 1.63^a^	66.52 ± 0.37^a^	66.83 ± 0.48^a^	4.16
*R*_pa0_	6.47 ± 0.67^a^	5.82 ± 0.49^a^	13.38 ± 2.17^a^	5.82 ± 0.49^b^	51.64	7.76 ± 0.28^a^	7.21 ± 0.11^a^	14.77 ± 0.01^a^	7.21 ± 0.11^b^	47.46
*R* ^2^	0.996	–	0.995	–	0.10	0.999	–	0.999	–	0

For a single plant, calculated and observed parameter values that exhibit a statistically significant difference (*p* < 0.05) are marked with distinct superscript letters. For instance, 0.127 ± 0.007^b^ and 0.296 ± 0.018^a^ signify a significant discrepancy. Conversely, values that do not show a statistically significant difference (*p* > 0.05) are denoted with the same superscript letter, indicating no significant variation. An example of this would be 58.12 ± 0.73^a^ and 57.80 ± 0.88^a^, which suggest no significant difference. Percentage differences (PD, %) = (Value derived from *A*_n_ *–C*_i_ curves *–* Value derived from *A*_n_–*C*_a_ curves) / Value derived from *A*_n_ *–C*_i_ curves × 100%. The PD value is represented by its absolute value. The unit of *A*_max_, *R*_p0_, *R*_pa0_ and *R*_day_ is μmol m^-2^ s^-1^; the unit of *Γ*, Γ_*_ and *C*_i,TPU_ is μmol mol^-1^. It is important to highlight that a comparative analysis has been conducted in **Table 3**, examining the parameters Γ_*_, *R*_p0_ and *R*_day_ between *A*_n_–*C*_a_ and *A*_n_–*C*_i_ curves to ascertain whether there are significant differences between these parameters.

### 3.4 A_n_–C_a_ and A_n_–C_i_ curves and their fitting using the Model I or Model II at 2% O_2_ concentration

**Figure 4:**
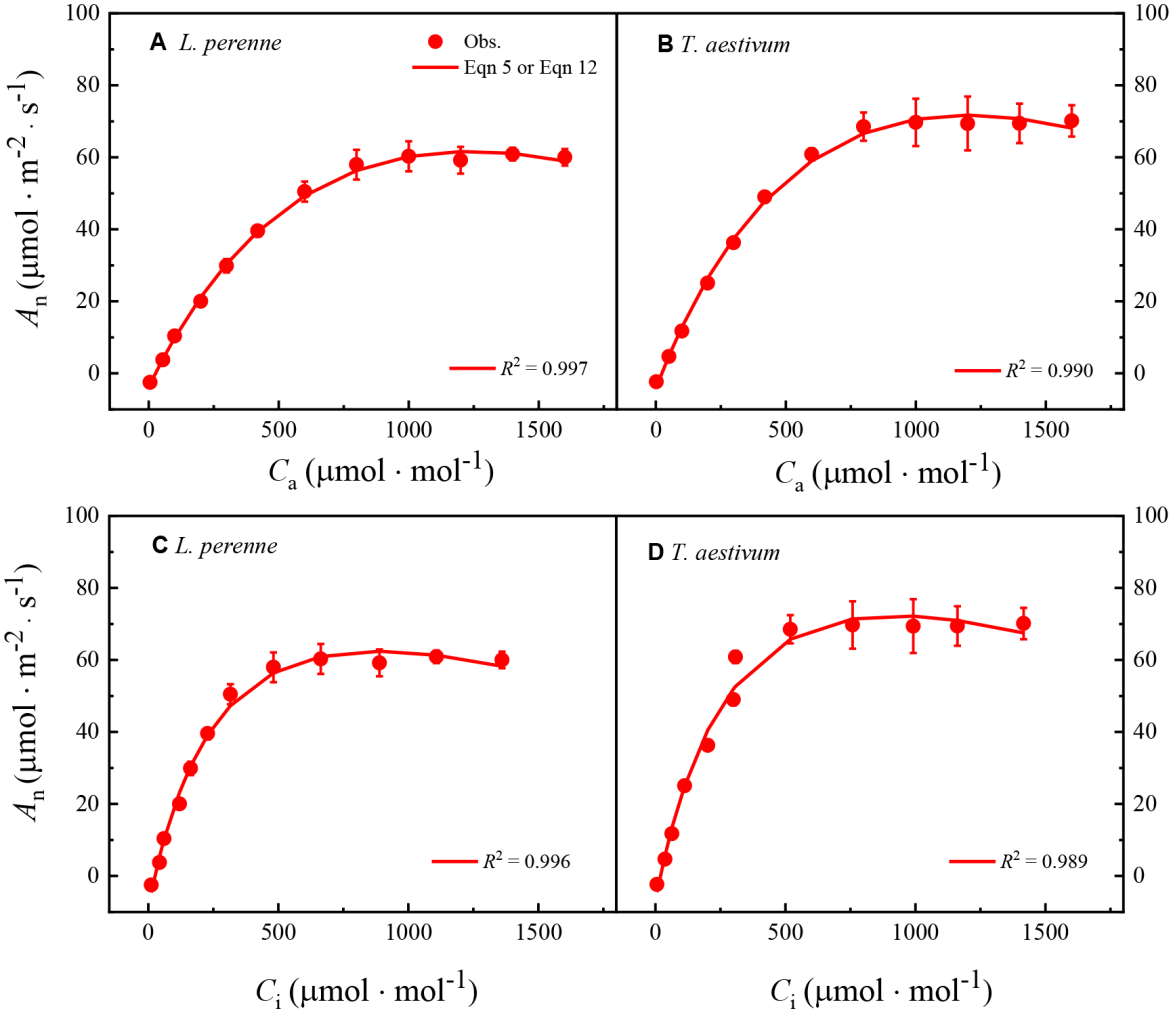
The *A*_n_–*C*_a_ and *A*_n_–*C*_i_ response curves for both *L. perenne* and *T. aestivum* under an oxygen concentration of 2%. These curves have been meticulously fitted using either Equations 5 or 12, which is indicated in the respective graphs. The solid red dots scattered across the curves correspond to the observed experimental data points, providing a visual representation of the actual measurements taken during the study. Each data point is presented as the mean value, with error bars indicating the standard error of the mean (SEM) to convey the variability within the dataset. It is important to note that the data are based on three replicates (*n* = 3), ensuring a robust statistical foundation for the analysis.

**Figure 4** shows the *A*_n_–*C*_a_ and *A*_n_–*C*_i_ curves of *L. perenne* and *T. aestivum* at 2% O_2_ concentration, along with the fitting curves generated by Model I or Model II, with the *R* ^2^ at least 0.989. As showed in **Figure 4**, both models can accurately reproduce the *A*_n_–*C*_a_ and *A*_n_–*C*_i_ curves of these two plants at 2% O_2_ concentration.

**Table 4** shows the parameters obtained by fitting the *A*_n_–*C*_a_ and *A*_n_–*C*_i_ curves of *L. perenne* and *T. aestivum* with Model I and Model II at 2% O_2_ concentration, including *α*_0_, *A*_max_, *C*_i,TPU_, *Γ*, Γ_*_, *R*_p0_, and *R*_pa0_. The data in **Table 4** indicate that, similar to the results in **Table 3**, for both the *A*_n_–*C*_a_ and *A*_n_–*C*_i_ curves, the *A*_max_ and *Γ* values obtained from fitting with the two models do not significantly differ (*p* > 0.05) from the corresponding observed values (except for the *Γ* values of *T. aestivum* obtained from the *A*_n_–*C*_a_ curve fitting with Model II). In addition, for *L. perenne*, the *R*_pa0_ value obtained by fitting the *A*_n_–*C*_a_ curve with Model II is close to the observed value, and there is no significant difference (*p* > 0.05) between the two (**Table 4**). On the contrary, when Model II is employed to fit the *A*_n_–*C*_i_ curves of *L. perenne* and *T. aestivum*, the *R*_pa0_ value obtained is significantly higher (*p* < 0.05) than the corresponding observed value (**Table 4**). Moreover, we found that there is a significant difference (*p* < 0.05) between the *R*_pa0_ value calculated by Model II and the observed data at 2% O_2_ concentration for *T. aestivum*. To avoid potential issues with the results, we opted to use Model II for fitting the *A*_n_–*C*_a_ curve within the range of 0 to 300 μmol·mol−¹. By doing this, the *R*_pa0_ value calculated with Model II is (2.45 ± 0.15) μmol·m−²·s−¹, which shows no significant difference when compared to the observed value of (2.31 ± 0.17) μmol·m−²·s−¹. In this instance, the coefficient of determination (*R* ^2^) exceeds 0.9995, signifying an exceptionally high level of agreement between the fitted and observed data points. For *L. perenne*, following the same procedures, we determined the *R*_pa0_ value using Model II to be (3.09 ± 0.38) μmol·m−²·s−¹. This value is in close proximity to the observed value, which is (3.03 ± 0.23) μmol·m−²·s−¹, with no significant divergence between them. When compared with the initial value of (3.68 ± 0.41) μmol·m−²·s−¹ presented in **Table 4**, the calculated value of (3.09 ± 0.38) μmol·m−²·s−¹ is more closely aligned with the observed values. Here as well, with an *R* ^2^ value surpassing 0.9977, we observe a remarkable alignment between the model’s predictions and the empirical data, reinforcing the model’s efficacy and reliability.

**Table 4:** The observed data and the outcomes generated by Model I and Model II for the two C_3_ species at a 2% O_2_ concentration for *A*_n_ *-C*_a_ and *A*_n_ *-C*_i_ curves are presented (mean ± *SE*, *n* = 3).

	*L. perenne*					*T. aestivum*				PD (%)
	*A*_n_ *-C*_a_		*A*_n_ *-C*_i_		PD (%)	*A*_n_ *-C*_a_		*A*_n_ *-C*_i_		
	Model I	Obs.	Model I	Obs.		Model I	Obs.	Model I	Obs.	
*α*_0_	0.150 ± 0.001^b^	^-^	0.410 ± 0.035^a^	^-^	63.41	0.189 ± 0.006^b^	^-^	0.392 ± 0.012^a^	^-^	51.79
*A*_max_	61.72 ± 1.92^a^	63.10 ± 2.07^a^	61.63 ± 1.75^a^	63.10 ± 2.07^a^	0.15	71.83 ± 3.43^a^	71.18 ± 3.44^a^	72.61 ± 3.72^a^	71.18 ± 3.44^a^	1.07
*C*_i,TPU_	1245.12 ± 28.85^a^	1266.55 ± 133.42^a^	821.27 ± 44.99^a^	895.56 ± 162.09^a^	51.61	1197.18 ± 6.35^a^	1400.58 ± 115.97^a^	846.74 ± 36.82^a^	1077.12 ± 190.73^a^	41.39
*Γ*	25.22 ± 3.02^a^	24.74 ± 3.53^a^	24.94 ± 2.61^a^	23.79 ± 3.65^a^	1.12	22.39 ± 0.98^a^	17.92 ± 1.11^b^	20.54 ± 0.81^a^	17.36 ± 1.17^a^	9.01
Γ_*_	17.70 ± 2.36^a^	–	21.84 ± 2.47^a^	–	18.96	16.35 ± 1.66^a^	–	15.61 ± 1.17^a^	–	4.74
*R*_p0_	2.60 ± 0.32^b^	–	7.45 ± 1.40^a^		65.10	3.05 ± 0.38^b^	–	5.86 ± 0.55^a^	–	47.95
*R*_day_	1.07 ± 0.08^b^	–	2.02 ± 0.17^a^	^-^	47.03	1.09 ± 0.11^b^	–	1.74 ± 0.13^a^	–	37.36
*R* ^2^	0.997	–	0.990	–	0.71	0.996	–	0.989	–	0.71
	A_n_-C_a_		A_n_-C_i_		PD (%)	A_n_-C_a_		A_n_-C_i_		PD (%)
	Model II	Obs.	Model II	Obs.		Model II	Obs.	Model II	Obs.	
*α*_0_	0.150 ± 0.001^b^	^-^	0.410 ± 0.035^a^	^-^	63.41	0.189 ± 0.006^b^	^-^	0.392 ± 0.012^a^	^-^	51.79
*A*_max_	61.72 ± 1.92^a^	63.10 ± 2.07^a^	61.63 ± 1.75^a^	63.10 ± 2.07^a^	0.15	71.83 ± 3.43^a^	71.18 ± 3.44^a^	72.61 ± 3.72^a^	71.18 ± 3.44^a^	1.07
*C*_i,TPU_	1245.12 ± 28.85^a^	1266.55 ± 133.42^a^	821.27 ± 44.99^a^	895.56 ± 162.09^a^	51.61	1197.18 ± 6.35^a^	1400.58 ± 115.97^a^	846.74 ± 36.82^a^	1077.12 ± 190.73^a^	41.39
*Γ*	25.22 ± 3.02^a^	24.74 ± 3.53^a^	24.94 ± 2.61^a^	23.79 ± 3.65^a^	1.12	22.39 ± 0.98^a^	17.92 ± 1.11^b^	20.54 ± 0.81^a^	17.36 ± 1.17^a^	9.01
*R*_pa0_	3.68 ± 0.41^a^	3.03 ± 0.23^a^	9.47 ± 1.56^a^	3.03 ± 0.23^b^	61.14	3.81 ± 0.33^a^	2.31 ± 0.17^b^	7.61 ± 0.44^a^	2.31 ± 0.17^b^	49.93
*R* ^2^	0.997	–	0.990	–	0.71	0.996	–	0.989	–	0.71

For a single plant, calculated and observed parameter values that exhibit a statistically significant difference (*p* < 0.05) are marked with distinct superscript letters. For instance, 0.150 ± 0.001^b^ and 0.410 ± 0.035^a^ signify a significant discrepancy. Conversely, values that do not show a statistically significant difference (*p* > 0.05) are denoted with the same superscript letter, indicating no significant variation. An example of this would be 61.72 ± 1.92^a^ and 63.10 ± 2.07^a^, which suggest no significant difference. Percentage differences (PD, %) = (Value derived from *A*_n_ *–C*_i_ curves - Value derived from *A*_n_–*C*_a_ curves) / Value derived from *A*_n_ *–C*_i_ curves × 100%. The PD value is represented by its absolute value. The unit of *A*_max_, *R*_p0_, *R*_pa0_ and *R*_day_ is μmol m^-2^ s^-1^; the unit of *Γ*, Γ_*_ and *C*_i,TPU_ is μmol mol^-1^. It is important to highlight that a comparative analysis has been conducted in **Table 4**, examining the parameters Γ_*_, *R*_p0_ and *R*_day_ between *A*_n_–*C*_a_ and *A*_n_–*C*_i_ curves to ascertain whether there are significant differences between these parameters.

It is noteworthy that there is a significant difference (*p* < 0.05) in the *α*_0_ values obtained by fitting the *A*_n_–*C*_a_ and *A*_n_–*C*_i_ curves of *L. perenne* and *T. aestivum* with the Model I. The values of *α*_0_ derived from *A*_n_ *–C*_a_ curves estimated by Model I were 63.41% and 51.79% lower than those obtained by fitting their *A*_n_–*C*_i_ curves for *L. perenne* and *T. aestivum* at 2% O_2_ conditions. This finding may indicate that the model is sensitive to different CO_2_ response curves. Furthermore, when comparing **Tables 2**, **4**, it can be observed that the Γ_*_ values derived from Model I are higher than those estimated by the FvCB model for both plant species.

## 4 Discussions

### 4.1 The FvCB model in fitting CO_2_-response curve of photosynthesis and estimating photosynthetic parameters

In this study, the application of the FvCB model and Model I highlighted the importance of model selection in simulating the process of photosynthesis in C_3_ plant species. The differences in the performance of the two models across different oxygen concentrations provided valuable information about the applicability and parameter sensitivity of these models.

The FvCB model is a widely used photosynthesis model in plant physiological research, based on the photosynthetic biochemical mechanism proposed by Farquhar et al. (1980). This model predicts the photosynthetic rate of plants under different environmental conditions by simulating key biochemical processes in photosynthesis, such as the carboxylation reaction of Rubisco, the regeneration of RuBP, and photorespiration. The FvCB model is particularly suitable for analyzing the photosynthetic performance of C_3_ plant species under changing environmental conditions, such as different CO_2_ concentrations, temperatures, and light intensities (von Caemmerer and Farquhar, 1981; Walker et al., 2017; Miyazawa et al., 2020; Xiao et al., 2021; Yin et al., 2021). In practical applications, the FvCB model has been used to assess the potential impact of climate change on crop yields (Long and Bernacchi, 2003; Fan et al., 2011) and to study plant adaptability to environmental changes (Morfopoulos et al., 2014; Han et al., 2020). For example, by simulating photosynthesis under different CO_2_ concentrations, researchers can predict the future impact of increasing atmospheric CO_2_ concentrations on plant growth and ecosystem carbon cycling (Ainsworth and Long, 2005, 2021). In addition, the FvCB model has also been used to optimize agricultural management practices, such as irrigation and fertilization, to improve the light energy utilization efficiency and yield of crops (Zhu et al., 2010; De Kauwe et al., 2016; Vijayakumar et al., 2024).

Although the FvCB model holds significant value in plant physiological ecology research, its accuracy and applicability are still constrained by the model parameterization method and changes in environmental conditions. For example, in this study, fitting the *A*_n_–*C*_a_ curves or *A*_n_–*C*_i_ curves of plants with the model produced five important photosynthetic parameters: *J*_A-max_, *V*_cmax_, *V*_TPU_, Γ_*_, and *R*_day_ (**Tables 1**, **2**). However, only the estimated parameter *J*_A-max_ can be directly compared with the observed *J*_f-max_ value (**Tables 1**, **2**). At the same time, under normal conditions, the distribution of the electron flow from Photosystem II clearly indicates that the *J*_A-max_ allocated for carbon assimilation is significantly lower than the *J*_f-max_. In the FvCB model, this parameter is not obtained by fitting the electron transport rate to the CO_2_ response (*J–C*_i_) data, but is indirectly estimated by fitting the *A*_n_–*C*_a_ curve or *A*_n_–*C*_i_ curve. This may lead to the model overestimating or underestimating the *J*_A-max_ in plants. In this study, it was found that the *J*_A-max_ value obtained by fitting the *A*_n_–*C*_a_ curve of *L. perenne* with the FvCB model was significantly lower (*p* < 0.05) than the observed value at 21% O_2_ concentration (**Table 1**), whereas the *J*_A-max_ value obtained by fitting the *A*_n_–*C*_i_ curve of *L. perenne* was close to the observed value (**Table 1**). In contrast, the *J*_A-max_ values obtained by fitting the *A*_n_–*C*_a_ and *A*_n_–*C*_i_ curves of *T. aestivum* with the FvCB model showed the opposite trend (**Table 1**). However, the *J*_A-max_ value derived from fitting the *A*_n_–*C*_i_ curve of *T. aestivum* is markedly higher than the observed *J*_f-max_ value, indicating a significant divergence between the estimated value and the empirical data (**Table 1**). Given the starkly contrasting outcomes for *J*_A-max_ when fitting the *A*_n_–*C*_a_ and *A*_n_–*C*_i_ curves with the FvCB model, it remains uncertain which response curve is more justified. This phenomenon may be related to the inaccurate simulation of the carboxylation reaction of the key enzyme Rubisco in photosynthesis by the model. Under the condition of 21% O_2_ concentration, the oxygenation of Rubisco may be overestimated, resulting in the model’s prediction of *J*_A-max_ being either too high or too low.

On the other hand, as explained by von Caemmerer (2000) and Long and Bernacchi (2003), under 21% O_2_ conditions, *J*_f_ supports not only *J*_A_ but also *J*_O_, *J*_Nit_, and *J*_MAP_. This relationship can be expressed as *J*_f_ = *J*_A_ + *J*_O_ + *J*_Nit_ + *J*_MAP_. Consequently, *J*_A-max_ must be less than *J*_f-max_. Under 2% O_2_ conditions, *J*_O_ can be neglected. In this case, the relationship can be expressed as *J*_f_ = *J*_A_ + *J*_Nit_ + *J*_MAP_, and *J*_A-max_ must still be less than *J*_f-max_. Based on this criterion, we observed that under 21% O_2_ concentration the *J*_A-max_ estimated by the FvCB model when fitting the *A*_n_–*C*_i_ curve of *T. aestivum* exceeds the *J*_f-max_ (**Table 1**). Taking into account that *J*_Nit_ and *J*_MAP_ are non-zero, we further found that the *J*_A-max_ estimated by the FvCB model when fitting both the *A*_n_–*C*_a_ and *A*_n_–*C*_i_ curves of *L. perenne* and *T. aestivum* also surpasses the *J*_f-max_ under 2% O_2_ concentration (**Table 2**).

Furthermore, the findings in **Table 1** reveal a pattern in the *V*_cmax_ values derived from the FvCB model when it is applied to the *A*_n_–*C*_a_ and *A*_n_–*C*_i_ curves of *L. perenne* and *T. aestivum*. Specifically, the *V*_cmax_ values obtained from fitting the *A*_n_–*C*_a_ curves are consistently lower than those derived from fitting the *A*_n_–*C*_i_ curves for both plant species. A similar trend is observed for the estimation of *R*_day_; that is, the *R*_day_ values derived from the *A*_n_–*C*_a_ curve fits are also consistently lower than those from the *A*_n_–*C*_i_ curve fits using the FvCB model for *L. perenne* and *T. aestivum*. Despite these observations, it remains challenging to discern which set of values—those from the *A*_n_–*C*_a_ or *A*_n_–*C*_i_ curve fits—provides a more accurate representation of the true *V*_cmax_ and *R*_day_ values for these two plant species. This uncertainty highlights the complexity of accurately modeling photosynthetic parameters. Thus, there is a need for further investigation to refine our understanding of how different models perform across various plant species and under different physiological conditions.

It is noteworthy that the choice between *A*_n_–*C*_a_ and *A*_n_–*C*_i_ curve fitting may be influenced by factors such as the atmospheric CO_2_ concentration, the specific photosynthetic pathway of the plant, and the presence of other environmental stressors. Therefore, a comprehensive analysis considering these factors and more experimental data may be necessary to determine the most appropriate model for accurately predicting *V*_cmax_ and *R*_day_ values. This could involve comparing the FvCB model’s predictive power with other models, examining its sensitivity to initial conditions, and assessing its robustness under varying environments.

At 2% oxygen concentration, the FvCB model’s fitting results differ significantly from those at the typical atmospheric concentration of 21% O_2_ (**Figures 1**, **2**; **Tables 1**, **2**). This discrepancy may indicate the plant’s adaptive responses in its photosynthetic machinery under hypoxic conditions. Notably, the model’s estimations of *J*_A-max_ for both *L. perenne* and *T. aestivum* closely approximate the actual observations, regardless of whether the *A*_n_–*C*_a_ curve or the *A*_n_–*C*_i_ curve is fitted (**Table 2**). It is important to highlight that the *J*_A-max_ value predicted by the FvCB model exceeds the observed *J*_f-max_ value for *T. aestivum* when either the *A*_n_–*C*_a_ curve or the *A*_n_–*C*_i_ curve is modeled. This outcome is perplexing given the prevailing photosynthetic theory as articulated by von Caemmerer (2000), which posits that *J*_A-max_ should be less than *J*_f-max_. Consequently, the findings are currently challenging to interpret.

Under hypoxic conditions of 2% O_2_, the carboxylation efficiency of Rubisco is likely impeded, and photorespiration gains prominence, as indicated by Busch and Sage (2017). The FvCB model reveals that Rubisco limitation, RuBP regeneration limitation, and TPU limitation, which are all pivotal to the photosynthetic process in C_3_ plants. Under low O_2_ environments, these limitations may become more pronounced and dampen the overall photosynthetic efficiency, as supported by Zhu et al. (2010). Furthermore, at 21% O_2_ (normoxic condition), the FvCB model-calculated *V*_cmax_ values for the *A*_n_–*C*_a_ curve are systematically lower than those for the *A*_n_–*C*_i_ curve when analyzing the two plant species (**Table 1**). This suggests that photosynthetic parameters are sensitive to oxygen levels, and the FvCB model may require refinements to accurately estimate them under different environmental conditions.

### 4.2 Model I and Model II in fitting CO_2_-response curve of photosynthesis and estimating photosynthetic parameters

In this study, both Model I and Model II exhibited a high *R* ^2^ when fitting the *A*_n_–*C*_a_ and *A*_n_–*C*_i_ curves of *L. perenne* and *T. aestivum*, indicating that the two models have high accuracy in simulating the photosynthesis curves under varying oxygen concentrations (**Figures 3**, **4**). This result may be attributed to the advantages of the Model I and Model II in parameterization and model structure, enabling them to better capture the photosynthetic response of plants under different environmental conditions. Moreover, both models effectively capture the *A*_n_–*C*_a_ and *A*_n_–*C*_i_ curves, demonstrating the reduction in carbon assimilation rates under elevated CO_2_ concentrations. They also enable the direct calculation of *C*_TPU_ (**Tables 3**, **4**). The results of our study show that the models’ estimation of the *A*_max_ and *Γ* values for *L. perenne* and *T. aestivum* under the two O_2_ concentrations does not show significant differences from the corresponding observed values (**Tables 3**, **4**). Indeed, a notable discrepancy is observed between the *R*_pa0_ values derived from fitting the *A*_n_–*C*_a_ curves and those derived from fitting the *A*_n_–*C*_i_ curves, as shown in **Tables 3**, **4**. Despite this, the scientific community has not yet reached a definitive conclusion on which of these *R*_pa0_ values—obtained from the *A*_n_–*C*_a_ curve or the *A*_n_–*C*_i_ curve—more accurately represents the true *R*_pa0_ value. However, considering the nuances of measurement technology, it is plausible to suggest that the *R*_pa0_ value obtained from the *A*_n_–*C*_a_ curve may be closer to the actual *R*_pa0_ value. This assumption is based on the belief that the *R*_pa0_ values, particularly those fitted for *L. perenne* and *T. aestivum* using Model II, reflect the true *R*_pa0_ values for these plant species. This belief is further supported by the *A*_n_–*C*_a_ curve’s more direct measurement of CO_2_ assimilation and its lesser influenced by internal CO_2_ concentration changes compared to the *A*_n_–*C*_i_ curve. Therefore, the *R*_pa0_ derived from the *A*_n_–*C*_a_ curve is often considered to be more representative of the plant’s true photorespiratory rate at CO_2_ concentrations approaching zero.

Furthermore, the rationale behind this preference is rooted in the technology used in plant photosynthesis measurement instruments. These instruments utilize non-diffusive infrared CO_2_ analysis technology to measure CO_2_ concentrations. This method takes advantage of the significant absorption of CO_2_ at a specific wavelength of infrared light, a characteristic not shared by O_2_. Consequently, it is advisable to use the *A*_n_–*C*_a_ curves derived from such measurements for quantitative studies of plant *R*_pa0_. Given this, when conducting research on *R*_pa0_ in plants, it is more logical to fit the *A*_n_–*C*_a_ curves using Model II rather than the *A*_n_–*C*_i_ curves. Model II’s curve- fitting approach better matches the capabilities of non-diffusive infrared CO_2_ analysis technology, making it a more suitable choice for accurate and reliable *R*_pa0_ assessments. In this study, another notable finding is that once Model I can determine the value of *R*_day_, it can also compute the value of *R*_p0_ (**Tables 3**, **4**). However, the precision of determining *R*_day_ presents a challenge, which in turn affects the accuracy of the derived *R*_p0_. Nevertheless, our research suggests that when *R*_day_ can be accurately ascertained, our methodology offers a viable approach for estimating *R*_p0_.

### 4.3 Comparative analysis of the FvCB model and the new models

The FvCB model and Model I/II represent fundamentally divergent paradigms in photosynthetic modeling. As a biochemical mechanistic framework, the FvCB model simulates enzymatic processes governing carbon assimilation (Farquhar et al., 1980), whereas Model I/II adopts an empirical approach emphasizing practical parameterization. This dichotomy reflects their core objectives: the FvCB model prioritizes biochemical fidelity, while Model I/II emphasizes operational simplicity and environmental adaptability.

Our comparative analysis demonstrates that the superior predictive performance of Models I/II in estimating *R*_pa0_ and critical photosynthetic parameters (*A*_max_, *Γ*, and *C*_TPU_) across oxygen gradients stems from their distinct structural architectures. The FvCB model’s dependence on rigid biochemical constraints–including fixed *R*_day_/*V*_cmax_ ratios (von Caemmerer, 2013; De Kauwe et al., 2016), stoichiometric electron requirements (4 e^-^ per CO_2_; Long and Bernacchi, 2003), and invariant photorespiratory CO_2_ release (0.5 mol/RuBP oxygenation; Farquhar et al., 1980)–introduces systematic biases. In contrast, Model I dynamically parameterizes *R*_day_ (Equation 5), while Model II integrates photorespiration into an apparent rate (*R*_pa_; Equation 14), effectively decoupling photorespiratory flux from predefined biochemical ratios. This innovation enables direct empirical estimation of *R*_pa0_ from gas exchange data, especially under hypoxic conditions (e.g., 2% O_2_) where photorespiration suppression exposes the FvCB model’s limitations. Specifically, the fixed *R*_day_/*V*_cmax_ assumption the FvCB model (von Caemmerer, 2013) leads to 46%-47% underestimation of respiratory activity compared to experimental data (**Tables 1**, **2**), whereas the new models’ dynamic parameterization achieves precise alignment with observations.

Methodologically, the FvCB model’s indirect estimation of *J*_A-max_ via *A*_n_–*C*_i_ curve fitting propagates errors from uncertain electron transport partitioning (Sharkey et al., 2007). Models I/II circumvent this limitation through direct quantification of *A*_max_, *Γ*, *C*_TPU_, and *R*_pa0_, eliminating error accumulation inherent to multi-step biochemical approximations. This distinction explains their robust performance across 2% and 21% O_2_ environments (**Figures 3**, **4**), demonstrating adaptability to oxygen fluctuations in both agricultural and natural ecosystems.

The FvCB model’s systematic underestimation of *V*_cmax_ and *R*_day_ during *A*_n_–*C*_a_ curve fitting further highlights its sensitivity to stomatal conductance dynamics—a confounding factor absent in *A*_n_–*C*_i_ analyses. Models I/II address this through unified *C*_a_-*C*_i_ treatment within a single analytical framework (Equations 5, 12), minimizing stomatal-induced artifacts. This proves critical under 2% O_2_, where stomatal closure amplifies discrepancies in FvCB-derived parameters. Additionally, the non-asymptotic formulation of Models I/II (Equations 5, 12) better captures *C*_TPU_ inflection points than the FvCB model’s segmented approach (**Figures 3**, **4**), which struggles to resolve RuBP- versus TPU-limited transitions under dynamic conditions.

However, these advancements come with trade-offs: Models I/II cannot estimate *V*_cmax_ or *J*_A-max_—parameters critical for Rubisco kinetic analyses (Farquhar et al., 1980; Bernacchi et al., 2013). This reflects the inherent tension between empirical accuracy and biochemical interpretability, necessitating context-specific model selection. Future hybrid frameworks could integrate Model I/II’s empirical strengths with the FvCB model’s biochemical resolution, particularly to disentangle photorespiratory and respiratory fluxes—a capability demonstrated in low-O_2_ environments where traditional partitioning assumptions fail (Xiong et al., 2022).

Enhanced predictive accuracy comes with increased parameterization complexity. Determining nuanced parameters like *R*_day_ requires high-precision gas exchange measurements and measured method under controlled conditions (Medlyn et al., 2011; Yin and Amthor, 2024), posing challenges for resource-limited studies. Furthermore, while coefficients *α*_c_/*α*_c1_, *β*_c_/*β*_c1_ and *γ*_c_/*γ*_c1_ in Models I/II are environment-dependent (Equations 5, 12), their biochemical basis—particularly regarding Rubisco carboxylation- oxygenation kinetics—remains unresolved. Clarifying these relationships through multi-omics approaches (e.g., concurrent chlorophyll fluorescence and metabolomic profiling) could bridge empirical models with photosynthetic biochemistry (Smith et al., 2023), transforming them into mechanistically robust tools for climate resilience research.

Furthermore, the limited sample size in our study may compromise the generalizability of our findings. Our experiments focused exclusively on two C_3_ plant species, *L. perenne* and *T. aestivum*. As such, additional validation is essential to establish the broader applicability of the new models to other plant species, particularly those with distinct photosynthetic pathways, such as C_4_ and CAM plants. Moreover, the models were evaluated under specific environmental conditions (2% and 21% O_2_ concentrations), and their performance under other stressors—such as high temperature, drought, or elevated CO_2_ levels—has yet to be fully explored. Additionally, to enhance the accuracy of *R*_pa0_ estimation, we recommend incorporating several additional measurement points at low CO_2_ concentrations (below 200 μmol·mol−¹), specifically at 30, 80, and 150 μmol·mol−¹. This refinement, however, necessitates increased time and effort to obtain comprehensive *A*_n_–*C*_a_ or *A*_n_–*C*_i_ curves.

## 5 Conclusions

In conclusion, this study underscores the essential requirement for accurate model parameters and their relevance when selecting photosynthesis models. It also highlights the significance of ongoing efforts to enhance these models to improve predictions of plant photosynthesis under diverse environmental conditions. Further exploration into the molecular mechanisms underlying Rubisco-catalyzed reactions and photorespiration is crucial, as it will not only refine model accuracy but also bolster our predictive capabilities in the face of environmental changes. Such advancements are pivotal to optimizing agricultural strategies and ecological preservation.

Moreover, both Model I and Model II have shown remarkable performance, particularly under varying oxygen levels, positioning them as valuable tools for analyzing C_3_ plant photosynthesis. Their consistent and reliable estimation of key parameters such as *A*_max_, *Γ*, and *R*_pa0_, coupled with their proficiency in fitting both *A*_n_–*C*_a_ and *A*_n_–*C*_i_ curves, offers a more precise depiction of plant photosynthetic mechanisms. As research progresses, future research should focus on validating these models across a broader range of temperatures, light intensities, and CO_2_ levels to enhance their robustness. Additionally, adapting these models for C_4_ and CAM plants by incorporating their unique biochemical pathways, such as PEPCase activity in C_4_ plants and temporal CO_2_ uptake in CAM plants, would significantly expand their applicability. Extensive validation across multiple species and conditions is essential to refine the models and ensure their accuracy. Comparative studies across different photosynthetic pathways will highlight areas for improvement and contribute to the development of generalized frameworks applicable across diverse plant types. Integrating these models into ecosystem models could also provide valuable insights into carbon cycling and ecosystem dynamics. Overall, although the new models are promising, more adaptation and validation are required to fully tap their potential in predicting photosynthetic responses among different plant species under various environmental conditions.

## Data Availability

The original contributions presented in the study are included in the article/**Supplementary Material**. Further inquiries can be directed to the corresponding authors.
