# Supplementary material for: Evaluating photosynthetic models and their potency in assessing plant responses to changing oxygen concentrations: a comparative analysis of A n–C a and A n–C i curves in Lolium perenne and Triticum aestivum
Source: Front Plant Sci. 2025 Apr 29;16:1575217. doi: 10.3389/fpls.2025.1575217 (PMC12069373; doi:10.3389/fpls.2025.1575217)
Supplement: Supplementary file 1 [file Table1.docx]

**Table S1** Definitions of the abbreviations

| Abbreviation | Definition | Units |
| --- | --- | --- |
| *A*_n_ | Net photosynthetic rate | μmol m^-2^ s^-1^ |
| *A*_n_–*C*_i_ | CO_2_-response curve of photosynthesis |  |
| *A*_n_–*I* | Light-response curve of photosynthesis |  |
| *A*_max_ | Maximum net photosynthetic rate | μmol m^-2^ s^-1^ |
| *C*_a_ | Atmospheric CO_2_ concentration | μmol mol^-1^ |
| *C*_i_ | Intercellular CO_2_ concentration | μmol mol^-1^ |
| *C* _TPU_ | *C*_i_ or *C*_a_ transit points from RuBP-limited to TPU-limited photosynthesis | μmol mol^-1^ |
| *g*_s_ | Stomatal conductance | mol m^-2^ s^-1^ |
| *g*_0_ | Minimum stomatal conductance | mol m^-2^ s^-1^ |
| *J*_A_ | Electron transport rate for C assimilation | μmol m^-2^ s^-1^ |
| *J*_O_ | Electron transport rate for photorespiratory rate | μmol mol^-1^ |
| *J*_Nit_ | Electron transport rate for reduction of nitrate to ammonium | μmol mol^-1^ |
| *J*_MAP_ | Electron transport rate for the light-driven oxygen uptake via the Mehler ascorbate peroxidase (MAP) reaction | μmol mol^-1^ |
| *J*_f_ | Electron transport rate from Photosystem II | μmol mol^-1^ |
| *J*_A-max_ | Maximum electron transport rate estimated by FvCB model | μmol m^-2^ s^-1^ |
| *J*_f-max_ | Maximum electron transport rate from Photosystem II | μmol m^-2^ s^-1^ |
| *h*_s_ | Relative humidity of the air |  |
| *I* | Light intensity | μmol m^-2^ s^-1^ |
| *m* | Empirical coefficient |  |
| *R*_day_ | Day respiratory rate | μmol m^-2^ s^-1^ |
| *R*_p0_ | Photorespiratory rate at *C*_a_ or *C*_i_ = 0 μmol mol^-1^ | μmol m^-2^ s^-1^ |
| *R*_pa0_ | Apparent photorespiratory rate at *C*_a_ or *C*_i_ = 0 μmol mol^-1^ | μmol m^-2^ s^-1^ |
| *V*_cmax_ | Maximum velocities of the carboxylase | μmol m^-2^ s^-1^ |
| *V*_TPU_ | Triose phosphate utilization rate | μmol m^-2^ s^-1^ |
| *Γ_*_* | Photorespiratory CO_2_ compensation point in the absence of *R*_day_ | μmol μmol ^-1^ |
| *Γ* | Photorespiratory CO_2_ compensation point in the presence of *R*_day_ | μmol μmol ^-1^ |
